# Janus Kinase Inhibitors for Alopecia Areata: A Systematic Review and Meta-Analysis

**DOI:** 10.1001/jamanetworkopen.2023.20351

**Published:** 2023-06-27

**Authors:** Ming Liu, Ya Gao, Yuan Yuan, Kelu Yang, Caiyi Shen, Jiancheng Wang, Jinhui Tian

**Affiliations:** 1 Evidence-Based Medicine Center, School of Basic Medical Sciences, Lanzhou University, Lanzhou, China; 2 Department of Health Research Methods, Evidence, and Impact, McMaster University, Hamilton, Ontario, Canada; 3 Department of Dermatology, Gansu Provincial Central Hospital, Lanzhou, China; 4 Department of Dermatology, Gansu Provincial Maternity and Child-care Hospital, Lanzhou, China; 5 Department of Public Health and Primary Care, Academic Centre for Nursing and Midwifery, Katholieke Universiteit Leuven, Leuven, Belgium; 6 The First School of Clinical Medicine, Lanzhou University, Lanzhou, China; 7 Department of Geriatrics, Gansu Provincial Hospital, Lanzhou, China; 8 Key Laboratory of Evidence-based Medicine and Knowledge Translation of Gansu Province, Lanzhou, China

## Abstract

**Importance:**

Alopecia areata (AA) is a common chronic tissue-specific autoimmune disease. Several studies have reported outcomes of Janus kinase (JAK) inhibitors for treating AA, but limited evidence has emerged.

**Objective:**

To evaluate the effectiveness and safety associated with JAK inhibitors for AA.

**Data Sources:**

MEDLINE, Embase, and CENTRAL (Cochrane Central Register of Controlled Trials) were searched from inception until August 2022.

**Study Selection:**

Only randomized clinical trials (RCTs) were included. Pairs of reviewers independently and in duplicate selected the studies.

**Data Extraction and Synthesis:**

Hartung-Knapp-Sidik-Jonkman random-effects models were used for meta-analysis. Certainty of evidence was evaluated using the Grading of Recommendations, Assessment, Development, and Evaluations (GRADE) approach. This study is reported according to the Preferred Reporting Items for Systematic Reviews and Meta-analyses (PRISMA) reporting guideline.

**Main Outcomes and Measures:**

The primary outcomes of interest were (1) proportion of patients who achieved 30%, 50%, and 90% improvement in Severity of Alopecia Tool (SALT) score from baseline, (2) change from baseline SALT score, and (3) treatment-related adverse event (AE).

**Results:**

Seven RCTs with 1710 patients (1083 females [63.3%]; mean [SD] age range, 36.3 [10.4] to 69.7 [16.2] years) were eligible and included in the study. JAK inhibitors were associated with more patients achieving 50% improvement (odds ratio [OR], 5.28 [95% CI, 1.69-16.46]; GRADE assessment: low certainty) and 90% improvement (OR, 8.15 [95% CI, 4.42-15.03]; GRADE assessment: low certainty) in SALT score from baseline compared with placebo. JAK inhibitors were associated with more lowered SALT scores from the baseline compared with placebo (mean difference [MD], –34.52 [95% CI, −37.80 to −31.24]; GRADE assessment: moderate certainty), and JAK inhibitors were not associated with more treatment-related AEs (relative risk [RR], 1.25 [95% CI, 1.00-1.57]; GRADE assessment: high certainty) compared with placebo. High certainty of evidence showed that JAK inhibitors may not be associated with more severe AEs compared with placebo (RR, 0.77; 95% CI, 0.41-1.43). The subgroup analysis showed that oral JAK inhibitors were more efficient than placebo (change from baseline SALT scores: MD, –36.80; 95% CI, −39.57 to −34.02), and no difference was found between external JAK inhibitors and placebo (change from baseline SALT scores: MD, –0.40; 95% CI, −11.30 to 10.50).

**Conclusions and Relevance:**

Results of this systematic review and meta-analysis suggest that JAK inhibitors, compared with placebo, were associated with hair regrowth and that the outcome of oral JAK inhibitors was better than the external route of administration. Although the safety and tolerability of JAK inhibitors were acceptable, longer RCTs are needed to further assess the effectiveness and safety of these treatments for AA.

## Key Points

**Question:**

What is the effectiveness and safety associated with Janus kinase (JAK) inhibitors for alopecia areata?

**Findings:**

In this systematic review and meta-analysis of 7 randomized clinical trials with 1710 patients, JAK inhibitors were associated with more hair regrowth compared with placebo, and the outcome of oral JAK inhibitors seemed better than the external route of administration.

**Meaning:**

These findings suggest that, although the safety and tolerability of JAK inhibitors were acceptable, longer trials are needed to further assess their effectiveness and safety for treatment of alopecia areata.

## Teaser

This systematic review and meta-analysis assesses outcomes from 7 randomized clinical trials, including changes in severity and adverse events, associated with use of JAK inhibitors among adults with alopecia areata.

## Introduction

Alopecia areata (AA) is a common chronic tissue-specific autoimmune disease that is characterized by nonscarring hair loss and preservation of the hair follicle.^1^ Several studies^2,3,4^ have confirmed that the Janus kinase (JAK)/signal transducer and activator of transcription signaling pathway in the initiation and progression of AA is a substantial factor in disease pathogenesis. These findings provide the rationale for the development and testing of JAK inhibitors to treat AA.^5,6^ There have been several case reports, clinical trials, and meta-analyses reporting outcomes of JAK inhibitors, such as tofacitinib, ruxolitinib, and baricitinib, for AA.^7,8,9^ However, the current evidence does not fully address the following questions: (1) is a JAK inhibitor better than placebo or other drugs for the treatment of AA, and is it safe?; (2) does the route of drug administration, oral vs topical, affect the outcome?; and (3) does the choice of a JAK inhibitor (tofacitinib vs ruxolitinib vs baricitinib) affect the therapeutic outcome? To address the current gaps in knowledge, we performed this systematic review and meta-analysis. We aimed to evaluate the effectiveness and safety associated with JAK inhibitors for AA.

## Methods

We conducted this study according to the Cochrane Handbook for Systematic Reviews of Interventions^10^ and reported it according to the Preferred Reporting Items for Systematic Reviews and Meta-analyses (PRISMA) reporting guideline.^11^ The study protocol was registered in PROSPERO (CRD42022354632).

### Eligibility Criteria, Search Strategy, and Study Selection

All randomized clinical trials (RCTs) that evaluated the efficacy and safety of both topical and systemic JAK inhibitors for AA were considered. There were no restrictions on the age or sex of patients, severity of AA, duration of AA episode, route of drug administration, or language of publication. Eligible trials must report at least 1 of the outcomes of interest.

We searched MEDLINE, Embase, and CENTRAL (Cochrane Central Register of Controlled Trials) from inception until August 2022. The search terms included *JAK inhibitors*, *Janus Kinase Inhibitors*, *ruxolitinib*, *tofacitinib*, *baracitinib*, *ritlecitinib*, *delgocitinib*, *alopecia areata*, *alopecia totalis*, *alopecia universalis*, and *randomized controlled trials*; eAppendix 1 in Supplement 1 provides the details of searches. We also searched the reference lists of relevant studies and review articles, and we performed forward and backward citation analyses using all databases in Web of Science from inception to August 1, 2022.

We screened, independently and in duplicate, the titles and abstracts as well as reviewed the full text of articles according to the eligibility criteria by using EndNote X8 software (Clarivate). We resolved disagreements by consensus and involved a third reviewer (J.T.) if necessary.

### Data Extraction

Pairs of reviewers (including M.L. and Y.G.) independently and in duplicate extracted data using standardized forms and resolved discrepancies by discussion and, if necessary, with adjudication by a third reviewer (J.T.). We collected (1) study information, including first author, publication year, design, trial registration, funding, country, and sample size; (2) patient characteristics, including diagnostic criteria of AA, age, sex, and duration of AA episode; (3) characteristics of interventions and comparators, including administered JAK inhibitors, dose, frequency, route of drug administration, treatment duration, and length of follow-up; and (4) data on each outcome of interest.

In the case of multiple records of the same trial, we collected all relevant data and analyzed them as a single study. In the case of discrepancies, we used the complete data set. Conversely, if a single record reported on more than 1 trial, we treated each trial as a separate study in the analysis.

### Outcomes and Risk-of-Bias Assessment

Primary outcomes of interest were (1) the proportion of patients achieving 30%, 50%, and 90% improvement in Severity of Alopecia Tool (SALT) score from baseline (SALT 30, SALT 50, and SALT 90), as rated by the patient’s medical practitioner; (2) change from baseline of SALT score, as rated by the patient’s medical practitioner; and (3) treatment-related adverse event (AE). Secondary outcomes of interest were the (1) percentage of patients achieving SALT scores of 10 or lower and 20 or lower during the treatment period, (2) percentage of patients with severe treatment-related AEs; (3) percentage of patients in discontinuation due to AEs.

Reviewers (including M.L. and Y.Y.) independently and in duplicate rated the risk of bias of each study on a per-outcome basis using a modified Cochrane risk-of-bias tool.^12^ This risk-of-bias tool included the following domains: random sequence generation; allocation concealment; blinding of patients, health care practitioners, data collectors, outcome assessors, and data analysts; incomplete outcome data; selective outcome reporting; and other sources of bias (eg, competing risks). Reviewers rated each domain as low risk of bias, probably low risk of bias, probably high risk of bias, or high risk of bias. We considered an outcome at increased risk of bias if at least 1 domain was rated as high or probably high risk of bias.

### Statistical Analysis

We analyzed outcomes according to the intention-to-treat principle; that is, all randomized patients were analyzed according to the initially assigned treatment group.^10^ We used the Hartung-Knapp-Sidik-Jonkman random-effects models for meta-analysis by R, version 3.6.3 (RStudio).^13^ For dichotomous outcomes, we calculated odds ratios (ORs) with 95% CIs for clinical effectiveness and relative risks (RRs) with 95% CIs for all AEs. For continuous outcomes, the mean differences (MDs) and 95% CIs were used to assess the credibility of the estimates.

We assessed the between-study heterogeneity with a visual inspection of forest plots and *I*^2^ statistics. We had planned that if 10 or more trials were available for an outcome, we would construct funnel plots to evaluate publication bias (ie, Harbord test for dichotomous outcomes, and Egger test for continuous outcomes).

If sufficient data were available (ie, at least 2 trials provided relevant information for each subgroup), we performed a prespecified subgroup analysis using the ICEMAN (Instrument to Assess the Credibility of Effect Modification Analyses) tool to evaluate the credibility of any apparent subgroup outcomes from route of drug administration (oral or external JAK inhibitors) and different drugs. To assess the robustness of results, we performed sensitivity analysis using the DerSimonian-Laird random-effect model, excluding high risk-of-bias outcomes and 0 events for both treatment groups.^14^

### Certainty of Evidence

The Grading of Recommendations, Assessment, Development, and Evaluations (GRADE) approach^15^ has been used to rate the overall certainty or quality of the evidence for each outcome on the basis of risk of bias,^16^ imprecision,^17^ indirectness,^18^ inconsistency,^19^ and publication bias.^20^ We used the GRADE approach to assess the certainty of evidence.

## Results

**Figure 1.**
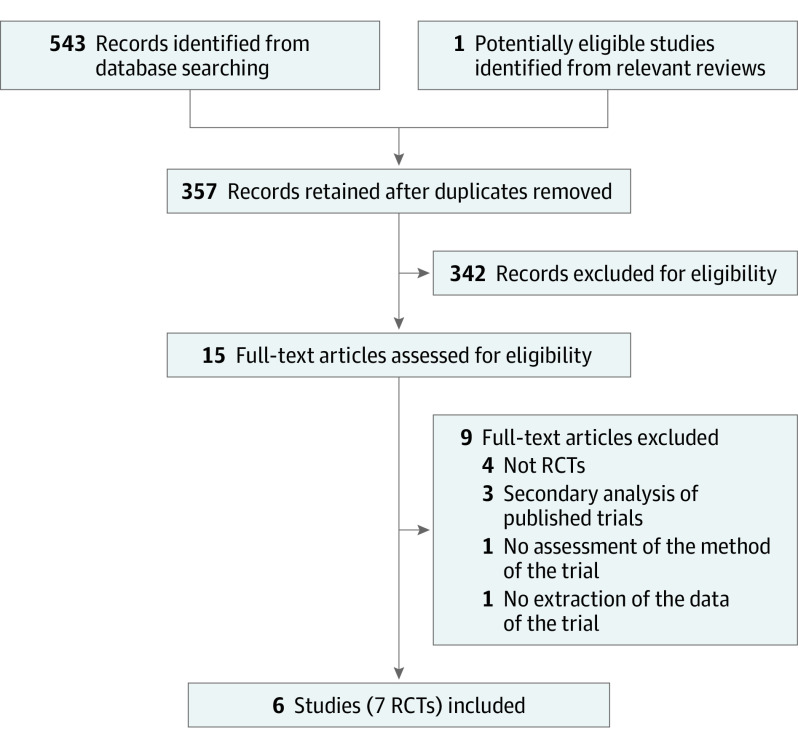
Flow Diagram of Study Selection RCT indicates randomized clinical trial.

The systematic search of the electronic databases and relevant reviews initially yielded 544 records, and 357 unique records remained after removing duplicates. After screening 357 titles and abstracts as well as 15 full texts, we ultimately included 7 RCTs reported in 6 studies^21,22,23,24,25,26^ (Figure 1).

Table 1 summarized the characteristics of the 7 eligible RCTs.^21,22,23,24,25,26^ The trials were published between 2019 and 2022 and were conducted in 11 countries. Among the 1710 patients included in the trials, the mean (SD) age range was 36.3 (10.4) to 69.7 (16.2) years; the mean (SD) duration of AA episode ranged from 2.3 (0.9) to 4.2 (1.3) years; and the total number of females was 1083 (63.3%) and of males was 627 (36.7%). The mean (SD) baseline SALT scores ranged from 59.5 (27.3) to 87.9 (17.4). All RCTs studied the efficacy and safety of JAK inhibitors compared with placebo, with external JAK inhibitors (ruxolitinib^22^ and delgocitinib^23^) in 2 RCTs and oral JAK inhibitors (ritlecitinib,^21^ brepocitinib,^21^ deuruxolitinib [CTP-543],^24^ and baricitinib^25,26^) in 5 RCTs. eTable 1 in Supplement 1 provides the inclusion and exclusion criteria for patients for all 7 RCTs.

**Table 1.** Characteristics of the 6 Eligible Studies

Source	Country	Phase	Age, mean (SD), y	Males, No. (%)	Females, No. (%)	Duration of AA episode, mean (SD), y	Baseline SALT score, mean (SD)	Route of drug administration	JAK inhibitor	Sample size, JAK inhibitors to placebo recipients	Outcomes
King et al,^21^ 2021^a^	Australia, Canada, US	2a	36.3 (10.4)	44 (31.0)	98 (69.0)	2.3 (0.7)	88.1 (17.2)	Oral	Ritlecitinib: 200 mg, every day, 4 wk +50 mg, every day, 20 wk; brepocitinib: 60 mg, every day, 4 wk +30 mg, every day, 20 wk	95:47	SALT 30, SALT 50, SALT 90, severe AE, discontinuation due to AE, change from baseline of SALT score
Olsen et al,^22^ 2020	US	2	43.4 (13.2)	27 (34.6)	51 (65.4)	2.4 (1.1)	59.5 (27.3)	External	Ruxolitinib: 1.5% cream, twice a day, 24 wk	39:39	SALT 50, SALT 90, treatment-related AE, severe AE, discontinuation due to AE
Mikhaylov et al,^23^ 2023	US	2a	69.7 (16.2)	12 (38.7)	19 (61.3)	NR	69.7 (24.7)	External	Delgocitinib: 30 mg/g, twice a day, 12 wk	20:11	SALT 50, treatment-related AE, severe AE, change from baseline of SALT score
King et al,^24^ 2022^a^	US	2	36.8 (9.5)	44 (29.5)	105 (70.5)	4.2 (1.3)	87.9 (17.4)	Oral	Deuruxolitinib: 4 mg/8 mg/12 mg, twice a day, 24 wk	105:44	SALT 50, SALT score ≤20, treatment-related AE, severe AE, discontinuation due to AE
King et al,^25^ 2021^b^	US, Japan	2	41.0 (18.3)	28 (25.4)	82 (74.6)	4.0 (0.9)	87.2 (17.3)	Oral	Baricitinib: 1 mg, every day, 16 wk; baricitinib: 2 mg/4 mg, every day, 36 wk	82:28	SALT 30, SALT 50, SALT 90, SALT score ≤20, SALT score ≤10, treatment-related AE, severe AE, change from baseline of SALT score
King et al,^26^ 2022^a^	US, Japan, Korea, Mexico, Puerto Rico	3	37.4 (11.5)	271 (41.4)	383 (58.6)	3.6 (1.7)	85.6 (18.0)	Oral	Baricitinib: 2 mg/4 mg, every day, 36 wk	465:189	SALT 90, SALT score ≤20, SALT score ≤10, treatment-related AE, severe AE, discontinuation due to AE, change from baseline of SALT score
King et al,^26^ 2022^b^	US, Argentina, Australia, Brazil, China, Israel, Japan, Korea, Puerto Rico	3	38.0 (16.1)	201 (36.8)	345 (63.2)	4.3 (2.1)	85.1 (18.0)	Oral	Baricitinib: 2 mg/4 mg, every day, 36 wk	390:156	SALT 90, SALT score ≤20, SALT score ≤10, treatment-related AE, severe AE, discontinuation due to AE, change from baseline of SALT score

Abbreviations: AA, alopecia areata; AE, adverse event; BRAVE-AA1/BRAVE-AA2 trials, A Study of Baricitinib (LY3009104) in Adults With Severe or Very Severe Alopecia Areata; JAK, Janus kinase; NR, not reported; SALT, Severity of Alopecia Tool.

^a^ Refers to BRAVE-AA1 trial.^26^

^b^ Refers to BRAVE-AA2 trial.^26^

eTable 2 in Supplement 1 shows the risk of bias for each outcome. The results showed that 9 outcomes from 2 RCTs^21,23^ were rated as having high risk of bias because of incomplete outcome data. We did not construct funnel plots to evaluate the publication bias because fewer than 10 RCTs were available for all outcomes.

### Outcomes of JAK Inhibitors

#### Change From Baseline SALT Scores

**Figure 2.**
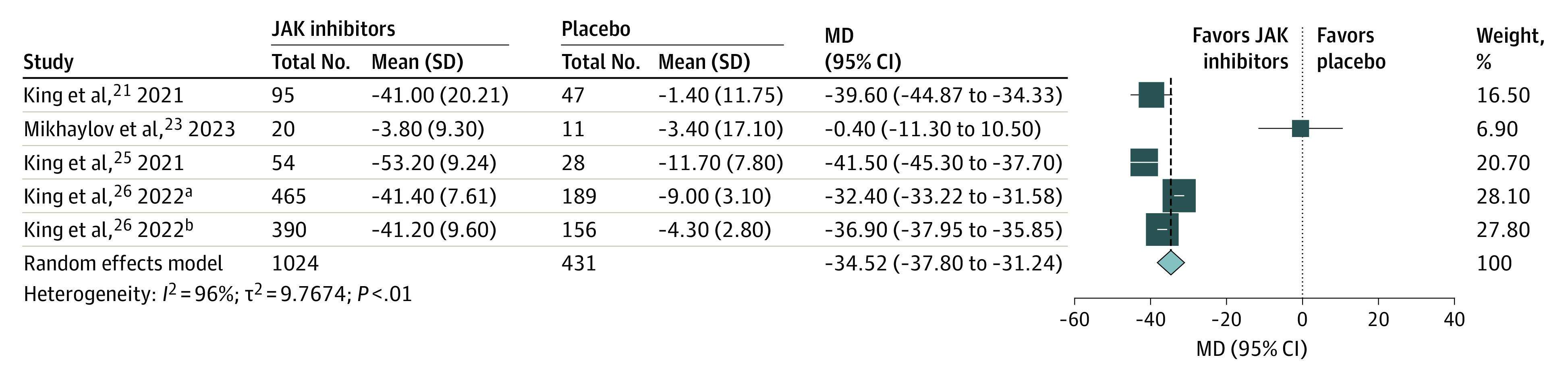
Janus Kinase (JAK) Inhibitors vs Placebo and Change From Baseline of Severity of Alopecia Tool Scores Size of the square denotes weighting, and diamond represents overall effect size. MD indicates mean difference. ^a^Refers to BRAVE-AA1 trial in King et al,^26^ 2022. ^b^Refers to BRAVE-AA2 trial in King et al,^26^ 2022.

Five trials,^21,23,25,26^ which included 1455 patients, reported on change from baseline SALT scores. The results showed that JAK inhibitors were associated with more lowered SALT scores from the baseline compared with placebo (MD, –34.52 [95% CI, −37.80 to −31.24]; *I*^2^ = 96%; GRADE assessment: moderate certainty) (Figure 2; Table 2).

**Table 2.** GRADE Summary of Findings for JAK Inhibitors vs Placebo Treatment for Alopecia Areata

Outcome	Results and measurements	Absolute effect size estimates		Difference	Certainty of evidence	Plain language summary
		Placebo	JAK inhibitors			
SALT 30	OR, 40.71 (95% CI, 7.62-217.41); based on data from 142 patients in 1 study^21^	21 per 1000	466 per 1000	445 per 1000 (95% CI, 119 to 936 per 1000)	Very low^a^	Whether JAK inhibitors were associated with a 30% improvement in SALT score remains uncertain
SALT 50	OR, 5.28 (95% CI, 1.69-16.46); based on data from 482 patients in 5 studies^21,22,23,24,25^	71 per 1000	288 per 1000	217 per 1000 (95% CI, 43 to 568 per 1000)	Low^b^	JAK inhibitors may be associated with 50% improvement in SALT score
SALT 90	OR, 8.15 (95% CI, 4.42-15.03); based on data from 1502 patients in 5 studies^21,22,25,26^	13 per 1000	97 per 1000	84 per 1000 (95% CI, 42 to 591 per 1000)	Low^c^	JAK inhibitors may be associated with 90% improvement in SALT score
SALT score ≤10	OR, 8.17 (95% CI, 4.42-15.08); based on data from 1282 patients in 3 studies^25,26^	13 per 1000	97 per 1000	84 per 1000 (95% CI, 42 to 591 per 1000)	Low^c^	JAK inhibitors may be associated with improvement in SALT score ≤10
SALT score ≤20	OR, 7.39 (95% CI, 4.82-11.33); based on data from 1431 patients in 4 studies^24,25,26^	66 per 1000	343 per 1000	277 per 1000 (95% CI, 188 to 379 per 1000)	Moderate^d^	JAK inhibitors probably were associated with improvement in SALT score ≤20
Treatment-related AE	RR, 1.25 (95% CI, 1.00-1.57); based on data from 1540 patients in 6 studies^22,23,24,25,26^	461 per 1000	576 per 1000	115 per 1000 (95% CI, −0 to 263 per 1000)	High	JAK inhibitors were associated with little or no difference in treatment-related AE
Severe AE	RR, 0.77 (95% CI, 0.41-1.43); based on data from 1682 patients in 7 studies RCTs^21,22,23,24,25,26^	26 per 1000	20 per 1000	−6 per 1000 (95% CI, −15 to 11 per 1000)	Moderate^d^	JAK inhibitors probably were associated with little or no difference in severe AE
Discontinuation due to AE	RR, 0.85 (95% CI, 0.42-1.71); based on data from 1596 patients in 5 studies^21,22,24,25^	26 per 1000	22 per 1000	−4 per 1000 (95% CI, −15 to 18 per 1000)	Moderate^d^	JAK inhibitors probably were associated with little or no difference in discontinuation due to AE
Change from baseline of SALT scores	Scale: 0-100; lower score was better; based on data from 1455 patients in 5 studies^21,23,25,26^	Mean, −4.3	Mean, −38.82	MD, −34.52 (95% CI, −37.80 to −31.24)	Moderate^e^	JAK inhibitors probably were associated with increased change from baseline of SALT scores

Abbreviations: AA, alopecia areata; AE, adverse event; GRADE, Grading of Recommendations, Assessment, Development, and Evaluations; JAK, Janus kinase; MD, mean difference; OR, odds ratio; RR, relative risk; SALT, Severity of Alopecia Tool.

^a^ Downgraded 1 level for serious risk of bias due to incomplete data, and downgraded 3 levels for extremely serious imprecision due to data from 1 study and wide CIs.

^b^ Downgraded 1 level for serious inconsistency due to statistical heterogeneity (*I*^2^ = 74%), and downgraded 1 level for serious imprecision due to wide CIs.

^c^ Downgraded 2 levels for very serious imprecision due to wide CIs.

^d^ Downgraded 1 level for serious imprecision due to wide CIs.

^e^ Downgraded 1 level for serious inconsistency due to statistical heterogeneity (*I*^2^ = 96%).

Subgroup analysis showed that oral JAK inhibitors were associated with more lowered SALT scores from the baseline compared with placebo (MD = –36.80; 95% CI, −39.57 to −34.02), and no difference was observed between external JAK inhibitors and placebo (MD = –0.40; 95% CI, −11.30 to 10.50). Subgroup analysis showed that ritlecitinib, brepocitinib, and baricitinib were associated with more lowered SALT scores from baseline compared with placebo, and no difference in outcome was found between delgocitinib and placebo (eAppendix 2 in Supplement 1).

#### SALT 30

One trial,^21^ including 142 patients, reported on SALT 30. The low certainty of evidence showed that JAK inhibitors were associated with more patients achieving a 30% improvement in SALT score from baseline compared with placebo (OR, 40.71; 95% CI, 7.62-217.41) (Table 2). However, there was only 1 event in the placebo group, and the certainty of evidence was very low.

#### SALT 50

**Figure 3.**
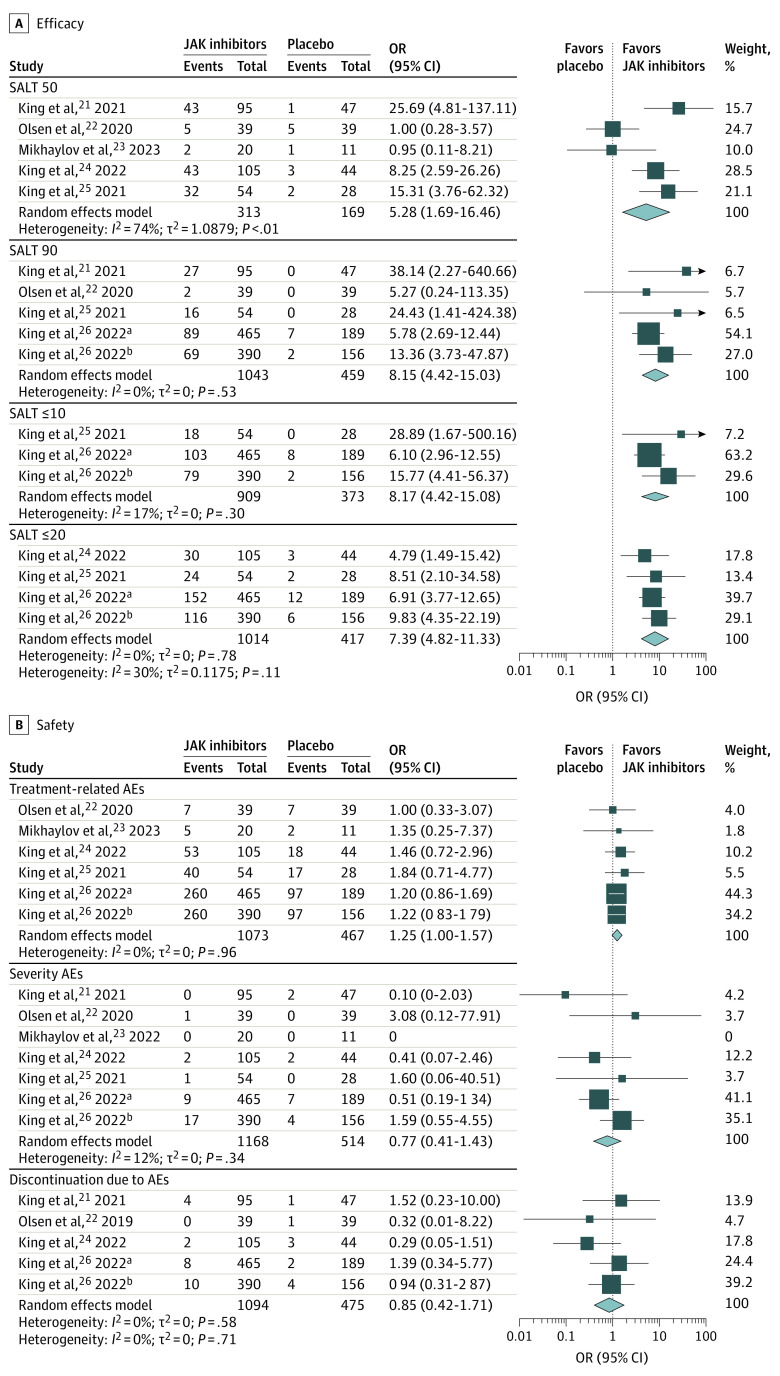
Janus Kinase (JAK) Inhibitors vs Placebo and Efficacy and Safety MD indicates mean difference; OR, odds ratio. ^a^Refers to BRAVE-AA1 trial in King et al,^26^ 2022. ^b^Refers to BRAVE-AA2 trial in King et al,^26^ 2022.

Five trials,^21,22,23,24,25^ including 482 patients, reported on SALT 50. The results showed that JAK inhibitors were associated with more patients achieving a 50% improvement in SALT score from baseline compared with placebo (OR, 5.28 [95% CI, 1.69-16.46]; *I*^2^ = 74%; GRADE assessment: low certainty) (Figure 3A; Table 2).

Subgroup analysis showed that oral JAK inhibitors were associated with more patients achieving a 50% improvement in SALT score from baseline compared with placebo, and no difference in outcome was found between external JAK inhibitors and placebo. Subgroup analysis also showed that ritlecitinib, brepocitinib, and baricitinib were associated with more patients achieving a 50% improvement in SALT score from baseline compared with placebo, and no difference in outcome was observed for ruxolitinib and delgocitinib compared with placebo (eAppendix 2 in Supplement 1).

#### SALT 90

Five trials,^21,22,25,26^ including 1502 patients, reported on SALT 90. The results showed that JAK inhibitors were associated with more patients achieving a 90% improvement in SALT score from baseline compared with placebo (OR, 8.15 [95% CI, 4.42-15.03]; *I*^2^ = 0%; GRADE assessment: low certainty) (Figure 3A; Table 2).

Subgroup analysis showed that oral JAK inhibitors were associated with more patients achieving a 90% improvement in SALT score from baseline compared with placebo, and no difference in outcome was observed between external JAK inhibitors and placebo. Subgroup analysis showed that ritlecitinib, brepocitinib, and baricitinib were associated with more patients achieving a 90% improvement in SALT score from baseline compared with placebo, and no difference was found for ruxolitinib (eAppendix 2 in Supplement 1).

#### SALT Scores 10 or Lower and 20 or Lower

Three trials,^25,26^ in which all JAK inhibitors were oral baricitinib and 1282 patients were included, reported on SALT scores of 10 or lower. The results showed that oral baricitinib was associated with a greater percentage of patients achieving SALT scores of 10 or lower over the treatment period compared with placebo (OR, 8.17 [95% CI, 4.42-15.08]; *I*^2^ = 17%; GRADE assessment: low certainty) (Figure 3A; Table 2).

Four trials,^24,25,26^ in which all JAK inhibitors were oral deuruxolitinib and baricitinib and 1431 patients were included, reported on SALT scores of 20 or lower. The results showed that oral deuruxolitinib and baricitinib were associated with a greater percentage of patients achieving SALT scores of 20 or lower over the treatment period compared with placebo (OR, 7.39 [95% CI, 4.82-11.33]; *I*^2^ = 0%; GRADE assessment: moderate certainty) (Figure 3A; Table 2). Subgroup analysis showed that deuruxolitinib and baricitinib were associated with more patients achieving SALT scores of 20 or lower over the treatment period compared with placebo (eAppendix 2 in Supplement 1).

### Adverse Events of JAK Inhibitors

Six trials,^22,23,24,25,26^ which included 1540 patients, reported on treatment-related AEs. The meta-analysis results showed that JAK inhibitors may not be associated with more treatment-related AEs compared with placebo (RR, 1.25 [95% CI, 1.00-1.57]; *I*^2^ = 0%; GRADE assessment: high certainty) (Figure 3B; Table 2).

Subgroup analysis showed that not all oral and external JAK inhibitors may be associated with more treatment-related AEs compared with placebo. Subgroup analysis showed that ruxolitinib, delgocitinib, deuruxolitinib, and baricitinib may not be associated with more treatment-related AEs compared with placebo (eAppendix 2 in Supplement 1).

Seven trials,^21,22,23,24,25,26^ including 1682 patients, reported on severe AE. The results showed that JAK inhibitors may not be associated with more severe AEs compared with placebo (RR, 0.77 [95% CI, 0.41-1.43]; *I*^2^ = 0%; GRADE assessment: high certainty) (Figure 3B; Table 2).

Subgroup analysis showed that not all oral and external JAK inhibitors may be associated with severe AEs compared with placebo. Subgroup analysis showed that ritlecitinib, brepocitinib, ruxolitinib, deuruxolitinib, and baricitinib may not be associated with more treatment-related AEs compared with placebo (eAppendix 2 in Supplement 1).

Five trials,^21,22,24,25^ including 1569 patients, reported on discontinuation due to AE. The results showed that JAK inhibitors may not be associated with more discontinuation due to AEs compared with placebo (RR, 0.85 [95% CI, 0.42-1.71]; *I*^2^ = 0%; GRADE assessment: high certainty) (Figure 3B; Table 2).

Subgroup analysis showed that not all oral and external JAK inhibitors may be associated with severe AEs compared with placebo. Subgroup analysis showed that ritlecitinib, brepocitinib, ruxolitinib, deuruxolitinib, and baricitinib may not be associated with more treatment-related AEs compared with placebo (eAppendix 2 in Supplement 1).

### Sensitivity Analysis

In the sensitivity analysis using the DerSimonian-Laird random-effect model (eFigures 14, 15, and 16 in Supplement 1), excluding high risk-of-bias outcomes (eFigures 17, 18, and 19 in Supplement 1) and 0 events for both groups, the results were similar to those in primary analysis (eFigures 20 and 21 in Supplement 1). Sensitivity analysis showed that the results of all analyses were reliable.

## Discussion

This study found that JAK inhibitors were associated with more patients achieving a 30%, 50%, or 90% improvement in SALT scores from baseline compared with placebo. JAK inhibitors were also associated with more SALT scores lowered from the baseline scores and had no association with more treatment-related AEs compared with placebo. Subgroup analysis found that oral JAK inhibitors were more efficient than placebo, and no difference was found between external JAK inhibitors and placebo. Simply, JAK inhibitors seemed to have resulted in more hair regrowth, and oral rather than external was a better route of administration.

A total number of 1710 patients were involved in the RCTs, and there were sufficiently large sample sizes for each outcome. The trials were conducted in 11 countries in the Americas, Australia, and Asia, which suggests that this study is representative in terms of population diversity. There was also clinical heterogeneity. However, this diversity may circumvent a potential concern for the reader: several of the trials^21,24,25,26^ were published by a group headed by an investigator named King. Although the King-led group seemed to dominate the AA field, the diversity in the included trials makes it clear to the reader that this is not an issue. The diversity also suggests that the evidence in this study has better clinical fitness.

Similar rates of males and females experience AA,^27^ but fewer cases in males than females were included in the study. Therefore, the results of this study may be more reliable when applied to female patients with AA. The patients with AA in this study were adults, at least 36 years of age; hence, the findings may not apply to children and adolescents.

In 2022, the US Food and Drug Administration approved baricitinib as treatment for adults with severe AA.^28^ In this study, the mean duration of AA episode ranged from 2.3 to 4.2 years. This finding indicates that JAK inhibitors were used more for the treatment of patients with severe AA, which is illustrated by the mean baseline SALT score ranging from 59.5 to 87.9 among patients in the included trials. Therefore, the results may be more appropriate in patients with severe AA and may work well in patients with early-onset or mild AA, but new clinical evidence is needed. Whether JAK inhibitors can be used in patients with nonsevere AA may be examined, along with the cost of treatment. Other drugs that cost much less than JAK inhibitors may have the same outcome for nonsevere AA.^29,30^ Hence, in light of this information, the applicability of the findings may be somewhat limited. It is necessary to update the study when new RCTs are published or to conduct more subgroup analyses or a network meta-analysis (NMA) when a trial’s data are sufficient.

Across the outcomes, the certainty of the evidence ranged from moderate to very low, and outcomes were downgraded for several reasons such as imprecision, incomplete data, and inconsistency. The levels of evidence for SALT 30, SALT 50, SALT 90, SALT score of 10 or lower, and SALT score of 20 or lower were downgraded because of very serious imprecision due to wide CIs. We believe that downgrading occurs because most of the evidence seems to be based on studies with 1 or 0 events. Therefore, more studies, larger sample sizes, and higher-level evidence are needed to facilitate further exploration.

We did not restrict the search by language, and we tried to contact the study authors to obtain additional data. However, we may have missed some unpublished data because we did not contact the drug manufacturers to obtain unpublished data. We will continue to monitor this area and will update the study if new data are released.

Regarding the outcomes of severe AE and discontinuation due to AE, there were 0 events involved. After referring to some of the latest analytic methods in meta-analysis,^31,32^ we excluded these 0-event studies and re-analyzed. The findings were robust to sensitivity analysis after excluding 0-event studies. The safety of JAK inhibitors was relatively reliable.

Several published systematic reviews have addressed the question of whether JAK inhibitors are effective and safe in AA and have reached different conclusions.^9,33,34,35,36^ A recent systematic review of 14 prospective studies,^34^ including 5 RCTs and 9 non-RCTs, found that recipients of oral JAK inhibitors had a higher reasonable response rate compared with control participants and that topical JAK inhibitors did not show any difference from power, and no difference was found in the risk of experiencing most types of AEs between recipients of JAK inhibitors vs placebo. Another systematic review^37^ also included 5 RCTs but was methodologically incomplete. In contrast, the present study included 7 RCTs and used more instruments to assess outcomes as well as the GRADE approach to rate the certainty of evidence, suggesting that the results are reliable and offer clinical guidance. In the context of the paucity of original studies in AA, the present study provides relatively high certainty of evidence to date.

### Limitations

This study has several limitations. First, it is well-established that patients with AA experience anxiety and depression, which can seriously affect quality of life.^38^ None of the included RCTs reported quality outcomes, which leads to a lack of evidence on quality of life in patients with AA. Therefore, we propose that a core outcome set for AA should be developed. Second, among the outcomes that were assessed, some were rated as having low certainty of evidence, mainly because of imprecision due to lack of evidence (eg, only 1 RCT) or a wide credible interval. We cannot change this finding and can only expect more trials with larger sample sizes to be published. Third, because of insufficient data, we were unable to perform the subgroup analysis as planned, and the credibility from the ICEMAN assessment was low for the completed subgroup analysis. Therefore, the effectiveness and safety of a specific oral JAK inhibitor could not be identified in this study. There is a strong need for an NMA in the future when sufficient data are available. Fourth, due to the lack of data in the included trials, we could not focus on the outcome of different drug doses and duration of treatment. Fifth, we did not take into account the economic cost and patient preferences throughout the study. Future studies are needed to address these issues.

## Conclusions

In this systematic review and meta-analysis, JAK inhibitors were associated with more hair regrowth, and oral JAK inhibitors may be better than external route of administration. Although the safety and tolerability of JAK inhibitors were acceptable, longer RCTs are required to further assess their effectiveness and safety. More trials with larger sample sizes would benefit future meta-analyses or NMAs and may help increase the certainty of evidence. The incomplete reporting of outcomes suggests the need for a core outcome set for AA.

## Notes

Supplement 1. **eAppendix 1.** Search Strategy for Each Database **eTable 1.** Inclusion and Exclusion Criteria of Patients With AA in Each RCTs **eTable 2.** Risk of Bias for Each Included RCTs **eAppendix 2.** Detail of Subgroup Analysis **eTable 3.** Credibility Assessment of Subgroup Analysis **eFigure 1.** Subgroup Analysis by Route of Administration of JAK Inhibitors Versus Placebo on Change From Baseline of SALT Scores **eFigure 2.** Subgroup Analysis by Different Drugs of JAK Inhibitors Versus Placebo on Change From Baseline of SALT Scores **eFigure 3.** Subgroup Analysis by Route of Administration of JAK Inhibitors Versus Placebo on SALT 50 **eFigure 4.** Subgroup Analysis by Different Drugs of JAK Inhibitors Versus Placebo on SALT 50 **eFigure 5.** Subgroup Analysis by Route of Administration of JAK Inhibitors Versus Placebo on SALT 90 **eFigure 6.** Subgroup Analysis by Different Drugs of JAK Inhibitors Versus Placebo on SALT 90 **eFigure 7.** Subgroup Analysis by Different Drugs of JAK Inhibitors Versus Placebo on SALT Score ≤20 **eFigure 8.** Subgroup Analysis by Route of Administration of JAK Inhibitors Versus Placebo on Treatment-related Adverse Event **eFigure 9.** Subgroup Analysis by Different Drugs of JAK Inhibitors Versus Placebo on Treatment-related Adverse Event **eFigure 10.** Subgroup Analysis by Route of Administration of JAK Inhibitors Versus Placebo on Severe Adverse Event **eFigure 11.** Subgroup Analysis by Different Drugs of JAK Inhibitors Versus Placebo on Severe Adverse Event **eFigure 12.** Subgroup Analysis by Route of Administration of JAK Inhibitors Versus Placebo on Discontinuation Due to Adverse Event **eFigure 13.** Subgroup Analysis by Different Drugs of JAK Inhibitors Versus Placebo on Discontinuation Due to Adverse Event **eFigure 14.** Sensitivity Analysis for Change From Baseline of SALT Scores Used the DerSimonian-Laird Random Effect Model **eFigure 15.** Sensitivity Analysis for SALT Scores (SALT 50, SALT 90, SALT Score ≤10 and SALT Score ≤20) Used the DerSimonian-Laird Random Effect Model **eFigure 16.** Sensitivity Analysis for Safety Used the DerSimonian-Laird Random Effect Model **eFigure 17.** Sensitivity Analysis for Change From Baseline of SALT Scores After Excluded High Risk Bias of Studies **eFigure 18.** Sensitivity Analysis for SALT Scores (SALT 50, SALT 90, SALT Score ≤10 and SALT Score ≤20) After Excluded High Risk Bias of Studies **eFigure 19.** Sensitivity Analysis for Safety After Excluded High Risk Bias of Studies **eFigure 20.** Sensitivity Analysis for SALT Scores (SALT 50, SALT 90, SALT Score ≤10 and SALT Score ≤20) After Excluded Zero-Events Studies **eFigure 21.** Sensitivity Analysis for Safety After Excluded Zero-Events Studies

Supplement 2.
Data Sharing Statement
