# Supplementary material for: Janus Kinase Inhibitors for Alopecia Areata: A Systematic Review and Meta-Analysis
Source: JAMA Netw Open. 2023 Jun 27;6(6):e2320351. doi: 10.1001/jamanetworkopen.2023.20351 (PMC10300710; doi:10.1001/jamanetworkopen.2023.20351)
Supplement: Supplement 1. — eAppendix 1. Search Strategy for Each Database eTable 1. Inclusion and Exclusion Criteria of Patients With AA in Each RCTs eTable 2. Risk of Bias for Each Included RCTs eAppendix 2. Detail of Subgroup Analysis eTable 3. Credibility Assessment of Subgroup Analysis eFigure 1. Subgroup Analysis by Route of Administration of JAK Inhibitors Versus Placebo on Change From Baseline of SALT Scores eFigure 2. Subgroup Analysis by Different Drugs of JAK Inhibitors Versus Placebo on Change From Baseline of SALT Scores eFigure 3. Subgroup Analysis by Route of Administration of JAK Inhibitors Versus Placebo on SALT 50 eFigure 4. Subgroup Analysis by Different Drugs of JAK Inhibitors Versus Placebo on SALT 50 eFigure 5. Subgroup Analysis by Route of Administration of JAK Inhibitors Versus Placebo on SALT 90 eFigure 6. Subgroup Analysis by Different Drugs of JAK Inhibitors Versus Placebo on SALT 90 eFigure 7. Subgroup Analysis by Different Drugs of JAK Inhibitors Versus Placebo on SALT Score ≤20 eFigure 8. Subgroup Analysis by Route of Administration of JAK Inhibitors Versus Placebo on Treatment-related Adverse Event eFigure 9. Subgroup Analysis by Different Drugs of JAK Inhibitors Versus Placebo on Treatment-related Adverse Event eFigure 10. Subgroup Analysis by Route of Administration of JAK Inhibitors Versus Placebo on Severe Adverse Event eFigure 11. Subgroup Analysis by Different Drugs of JAK Inhibitors Versus Placebo on Severe Adverse Event eFigure 12. Subgroup Analysis by Route of Administration of JAK Inhibitors Versus Placebo on Discontinuation Due to Adverse Event eFigure 13. Subgroup Analysis by Different Drugs of JAK Inhibitors Versus Placebo on Discontinuation Due to Adverse Event eFigure 14. Sensitivity Analysis for Change From Baseline of SALT Scores Used the DerSimonian-Laird Random Effect Model eFigure 15. Sensitivity Analysis for SALT Scores (SALT 50, SALT 90, SALT Score ≤10 and SALT Score ≤20) Used the DerSimonian-Laird Random Effect Model eFigure 16. Sensitivity [file jamanetwopen-e2320351-s001.pdf]

Supplemental Online Content

Liu M, Gao Y, Yuan Y, et al. Janus kinase inhibitors for alopecia areata: a systematic review and meta-analysis. *JAMA Netw Open*. 2023;6(6):e2320351. doi:10.1001/jamanetworkopen.2023.20351

eAppendix 1. Search Strategy for Each Database

eTable 1. Inclusion and Exclusion Criteria of Patients With AA in Each RCTs

eTable 2. Risk of Bias for Each Included RCTs

eAppendix 2. Detail of Subgroup Analysis

eTable 3. Credibility Assessment of Subgroup Analysis

eFigure 1. Subgroup Analysis by Route of Administration of JAK Inhibitors Versus Placebo on Change From Baseline of SALT Scores

eFigure 2. Subgroup Analysis by Different Drugs of JAK Inhibitors Versus Placebo on Change From Baseline of SALT Scores

eFigure 3. Subgroup Analysis by Route of Administration of JAK Inhibitors Versus Placebo on SALT 50

eFigure 4. Subgroup Analysis by Different Drugs of JAK Inhibitors Versus Placebo on SALT 50

eFigure 5. Subgroup Analysis by Route of Administration of JAK Inhibitors Versus Placebo on SALT 90

eFigure 6. Subgroup Analysis by Different Drugs of JAK Inhibitors Versus Placebo on SALT 90

eFigure 7. Subgroup Analysis by Different Drugs of JAK Inhibitors Versus Placebo on SALT Score ≤ 20

eFigure 8. Subgroup Analysis by Route of Administration of JAK Inhibitors Versus Placebo on Treatment-related Adverse Event

eFigure 9. Subgroup Analysis by Different Drugs of JAK Inhibitors Versus Placebo on Treatment-related Adverse Event

eFigure 10. Subgroup Analysis by Route of Administration of JAK Inhibitors Versus

© 2023 Liu M et al. *JAMA Network Open*

Placebo on Severe Adverse Event

eFigure 11. Subgroup Analysis by Different Drugs of JAK Inhibitors Versus Placebo on Severe Adverse Event

eFigure 12. Subgroup Analysis by Route of Administration of JAK Inhibitors Versus Placebo on Discontinuation Due to Adverse Event

eFigure 13. Subgroup Analysis by Different Drugs of JAK Inhibitors Versus Placebo on Discontinuation Due to Adverse Event

eFigure 14. Sensitivity Analysis for Change From Baseline of SALT Scores Used the DerSimonian-Laird Random Effect Model

eFigure 15. Sensitivity Analysis for SALT Scores (SALT 50, SALT 90, SALT Score ≤ 10 and SALT Score ≤ 20) Used the DerSimonian-Laird Random Effect Model

eFigure 16. Sensitivity Analysis for Safety Used the DerSimonian-Laird Random Effect Model

eFigure 17. Sensitivity Analysis for Change From Baseline of SALT Scores After Excluded High Risk Bias of Studies

eFigure 18. Sensitivity Analysis for SALT Scores (SALT 50, SALT 90, SALT Score ≤ 10 and SALT Score ≤ 20) After Excluded High Risk Bias of Studies

eFigure 19. Sensitivity Analysis for Safety After Excluded High Risk Bias of Studies

eFigure 20. Sensitivity Analysis for SALT Scores (SALT 50, SALT 90, SALT Score ≤ 10 and SALT Score ≤ 20) After Excluded Zero-Events Studies

eFigure 21. Sensitivity Analysis for Safety After Excluded Zero-Events Studies

This supplemental material has been provided by the authors to give readers additional information about their work.

© 2023 Liu M et al. *JAMA Network Open*

eAppendix 1: Search strategy for each database

Ovid MEDLIAN(R) ALL [1946 to Present]

1 exp alopecia areata/

2 (alopecia areata or alopecia circumscripta or alopecia totalis or alopecia universalis or alopecia celsi or pelade* or nonscarring hair loss or scarring hair loss).mp.

3 1 or 2

4 exp Janus Kinase Inhibitors/

5 (janus kinase inhibitors or janus kinase inhibitor or JAK Inhibitors or JAK inhibitor).mp.

6 exp ruxolitinib/

7 (ruxolitinib* or INCB-18424 or INCB018424 or INCB-018424 or INC-424 or INC424 or INCA24).mp.

8 exp tofacitinib/

9 (tofacitinib* or Xeljanz or CP 690,550 or CP 690550 or CP690550 or CP-690550 or CP-690,550).mp.

10 exp delgocitinib/

11 (baracitinib* or baricitinib* or delgocitinib* or ritlecitinib* or brepocitinib* or CTP-543).mp.

12 or/4-7

13 3 and 12

14 randomized controlled trial.pt.

15 controlled clinical trial.pt.

16 randomized.ab.

17 placebo.ab.

18 rug therapy.fs.

19 randomly.ab.

20 trial.ti.

21 groups.ab.

22 or/14-21

23 (animals not (humans and animals)).sh.

© 2023 Liu M et al. *JAMA Network Open*

24 22 not 23

25 13 and 24

EMBASE[1974 to August 2022]

1 exp alopecia areata/

2 (alopecia areata or alopecia circumscripta or alopecia totalis or alopecia universalis or alopecia celsi or pelade* or nonscarring hair loss or scarring hair loss).mp.

3 1 or 2

4 exp Janus Kinase Inhibitors/

5 (janus kinase inhibitors or janus kinase inhibitor or JAK Inhibitors or JAK inhibitor).mp.

6 exp ruxolitinib/

7 (ruxolitinib* or INCB-18424 or INCB018424 or INCB-018424 or INC-424 or INC424 or INCA24).mp.

8 exp tofacitinib/

9 (tofacitinib* or Xeljanz or CP 690,550 or CP 690550 or CP690550 or CP-690550 or CP-690,550).mp.

10 exp delgocitinib/

11 (baracitinib* or baricitinib* or delgocitinib* or ritlecitinib* or brepocitinib* or CTP-543).mp.

12 or/4-7

13 3 and 12

14 Randomized controlled trial/

15 Controlled clinical study/

16 random$.ti,ab.

17 randomization/

18 intermethod comparison/

19 placebo.ti,ab.

20 (compare or compared or comparison).ti.

21 ((evaluated or evaluate or evaluating or assessed or assess) and (compare or

© 2023 Liu M et al. *JAMA Network Open*

compared or comparing or comparison)).ab.

22 (open adj label).ti,ab.

23 ((double or single or double or singly) adj (blind or blinded or blindly)).ti,ab.

24 double blind procedure/

25 parallel group$1.ti,ab.

26 (crossover or cross over).ti,ab.

27 ((assign$ or match or matched or allocation) adj5 (alternate or group$1 or intervention$1 or patient$1 or subject$1 or participant$1)).ti,ab.

28 (assigned or allocated).ti,ab.

29 (controlled adj7 (study or design or trial)).ti,ab.

30 (volunteer or volunteers).ti,ab.

31 human experiment/

32 trial.ti.

33 or/14-32

34 (random$ adj sampl$ adj7 ("cross section$" or questionnaire$1 or survey$ or database$1)).ti,ab. not (comparative study/ or controlled study/ or randomi?ed controlled.ti,ab. or randomly assigned.ti,ab.)

35 Cross-sectional study/ not (randomized controlled trial/ or controlled clinical study/ or controlled study/ or randomi?ed controlled.ti,ab. or control group$1.ti,ab.)

36 (((case adj control$) and random$) not randomi?ed controlled).ti,ab.

37 (Systematic review not (trial or study)).ti.

38 (nonrandom$ not random$).ti,ab.

39 "Random field$".ti,ab.

40 (random cluster adj3 sampl$).ti,ab.

41 (review.ab. and review.pt.) not trial.ti.

42 "we searched".ab. and (review.ti. or review.pt.)

43 "update review".ab.

44 (databases adj4 searched).ab.

45 (rat or rats or mouse or mice or swine or porcine or murine or sheep or lambs or

© 2023 Liu M et al. *JAMA Network Open*

pigs or piglets or rabbit or rabbits or cat or cats or dog or dogs or cattle or bovine or monkey or monkeys or trout or marmoset$1).ti. and animal experiment/

46 Animal experiment/ not (human experiment/ or human/)

47 or/34-46

48 54 not 47

49 13 and 33 and 48

Cochrane Controlled Register of Trials

1 exp alopecia areata/

2 (alopecia areata or alopecia circumscripta or alopecia totalis or alopecia universalis or alopecia celsi or pelade* or nonscarring hair loss or scarring hair loss).mp.

3 1 or 2

4 exp Janus Kinase Inhibitors/

5 (janus kinase inhibitors or janus kinase inhibitor or JAK Inhibitors or JAK inhibitor).mp.

6 exp ruxolitinib/

7 (ruxolitinib* or INCB-18424 or INCB018424 or INCB-018424 or INC-424 or INC424 or INCA24).mp.

8 exp tofacitinib/

9 (tofacitinib* or Xeljanz or CP 690,550 or CP 690550 or CP690550 or CP-690550 or CP-690,550).mp.

10 exp delgocitinib/

11 (baracitinib* or baricitinib* or delgocitinib* or ritlecitinib* or brepocitinib* or CTP-543).mp.

12 or/4-7

13 3 and 12

© 2023 Liu M et al. *JAMA Network Open*

eTable 1. Inclusion and exclusion criteria of patients with AA in each RCTs

Author, year	Inclusion criteria	Exclusion criteria
King 2021a	Adults at least 18 years of age qualified for inclusion if they had AA with ≥ 50% scalp hair loss, no hair regrowth within 6 months of the screening and baseline visits, and a current episode of fixed hair loss of 7 years or less in duration.	Patients were excluded if they had another type of alopecia or active inflammatory disease involving the scalp, or if they used an oral or topical JAK inhibitor within 12 weeks of the first dose of the study drug, a biologic within 12 weeks or 5 half-lives (whichever is longer), systemic or intralesional treatment that could affect AA within 8 weeks or 5 half-lives, phototherapy within 4 weeks, or a topical treatment that could affect AA within 2 weeks.
Olsen 2019	Patients aged 18 through 70 years with a severity of alopecia tool score at baseline of 25% to 99% scalp hair loss and either a positive hair pull test or presence of exclamation mark hairs. The washout periods for all agents used to treat AA was 7 days before baseline for over-the-counter, nonprescription medications and 14 days for any prescription medications.	Key exclusion criteria included AT, AU, or exclusively ophiasis; less than 12 months of 25% to less than 50% hair loss and less than 6 months of 50% to 99% hair loss; evidence of diffuse, spontaneous terminal hair regrowth; history of spontaneous remission in the previous 2 years; and cytopenias at screening.
Mikhaylov 2022	Age ranged from 18 to 65 years, no restrictions on gender. Subjects must have signed and dated informed consent after receiving verbal and written information about the clinical trial. Subjects with unequivocal clinical diagnosis of moderate to severe scalp alopecia areata (patch type, totalis, universalis), as determined by the (sub) investigator, affecting a minimum of 30% scalp area at Visit 1 (Screening) and Visit 2 (Day 1, baseline);3.Minimum 6 month duration of hair loss at Visit 1 (Screening). No upper limit time limit. Subject must accept to not cut hair in the treated scalp areas during the trial.	Females who are pregnant or are breast feeding. Current signs of spontaneous hair regrowth. Diffuse type alopecia areata. Co-existing moderate to severe androgenic alopecia (Norwood-Hamilton stage IV-VI and Ludwig stage II and III). Subjects with changed or expected changes in medication for thyroid disease within 6 month before Visit 1 (screening) or during the trial. Systemic treatment with immunosuppressive drugs (e.g., methotrexate, cyclosporine, azathioprine), chloroquin derivatives, corticosteroids, or any other systemic therapy that in the opinion of the investigator could affect hair regrowth, within 6 weeks prior to randomization (inhaled or intra-nasal steroids corresponding to up to 1 mg prednisone for asthma or rhinitis may be used).

© 2023 Liu M et al. *JAMA Network Open*

King 2022a	18 and 65 years of age and were experiencing current episodes of hair loss because of AA lasting ≥6 months and not exceeding 10 years. The patients had ≥50% hair loss, as measured by the Severity of Alopecia Tool (SALT), at screening and baseline and were not concurrently being treated for AA or receiving other treatments that might have affected hair regrowth or immune responses.	Patients were excluded if they had received any systemic immunosuppressive medications (eg, methotrexate, cyclosporin, JAK inhibitors, etc.) within 3 months of screening or any biologic medications (eg., adalimumab or ustekinumab) within 6 months of screening.
King 2021b	Eligibility criteria included patients aged ≥18 years to ≤60 years for males and ≥18 years to ≤70 years for females with severe (Severity of Alopecia Tool [SALT] score of 50-94% scalp hair loss) or very severe AA (SALT score 95-100%). Male or nonpregnant, nonbreastfeeding female participants.	Primarily "diffuse" type of AA. Are currently experiencing other forms of alopecia or any other concomitant conditions that would interfere with evaluations of the effect of study medication on AA. Previously treated with an oral Janus kinase (JAK) inhibitor and had an inadequate response (for example, absence of significant terminal hair growth after at least 12 weeks of treatment).
King 2022b BRAVE-AA1	Eligibility criteria included patients aged ≥18 years to ≤60 years for males and ≥18 years to ≤70 years for females with severe (Severity of Alopecia Tool [SALT] score of 50-94% scalp hair loss) or very severe AA (SALT score 95-100%). Male or nonpregnant, nonbreastfeeding female participants.	Primarily "diffuse" type of AA. Are currently experiencing other forms of alopecia or any other concomitant conditions that would interfere with evaluations of the effect of study medication on AA. Previously treated with an oral Janus kinase (JAK) inhibitor and had an inadequate response (for example, absence of significant terminal hair growth after at least 12 weeks of treatment).
King 2022b BRAVE-AA2	Are at least 18 years and ≤60 years for males (≤70 years of age for females) at the time of informed consent. Have severe or very severe AA, as determined by all of the following: Current AA episode of more than 6 months' duration and hair loss encompassing ≥50% of the scalp, as measured by SALT (AA-IGA of 3 or 4) at screening and baseline; No spontaneous improvement over the past 6 months; Current episode of severe or very severe AA of less than 8 years. Note: participants who have severe or very severe AA for ≥8 years may be enrolled if episodes of regrowth, spontaneous or under treatment, have been observed on the affected areas over the past 8 years. Male or nonpregnant, nonbreastfeeding female participants.	Primarily "diffuse" type of AA. Are currently experiencing other forms of alopecia or any other concomitant conditions that would interfere with evaluations of the effect of study medication on AA. Previously treated with an oral Janus kinase (JAK) inhibitor and had an inadequate response (for example, absence of significant terminal hair growth after at least 12 weeks of treatment).

© 2023 Liu M et al. *JAMA Network Open*

© 2023 Liu M et al. *JAMA Network Open*

eTable 2. Risk of bias for each included RCTs

Study	Outcome	Random sequence generation	Allocation concealment	Blinding of patients	Blinding of health care providers	Blinding of data collectors	Blinding of outcome assessors/ adjudicators	Blinding of data analysts	Incomplete outcome data	Selective outcome reporting	Other bias
King 2021a	SALT 30	Definitely Low	Definitely Low	Definitely Low	Definitely Low	Definitely Low	Definitely Low	Definitely Low	Probably High	Probably Low	Definitely Low
King 2021a	SALT 50	Definitely Low	Definitely Low	Definitely Low	Definitely Low	Definitely Low	Definitely Low	Definitely Low	Probably High	Probably Low	Definitely Low
King 2021a	SALT 90	Definitely Low	Definitely Low	Definitely Low	Definitely Low	Definitely Low	Definitely Low	Definitely Low	Probably High	Probably Low	Definitely Low
King 2021a	Severe adverse event	Definitely Low	Definitely Low	Definitely Low	Definitely Low	Definitely Low	Definitely Low	Definitely Low	Probably High	Probably Low	Definitely Low
King 2021a	Discontinuation due to Adverse Event	Definitely Low	Definitely Low	Definitely Low	Definitely Low	Definitely Low	Definitely Low	Definitely Low	Probably High	Probably Low	Definitely Low
King 2021a	Change from baseline of SALT score	Definitely Low	Definitely Low	Definitely Low	Definitely Low	Definitely Low	Definitely Low	Definitely Low	Probably High	Probably Low	Definitely Low
Olsen 2019	SALT 50	Probably Low	Probably Low	Definitely Low	Definitely Low	Probably Low	Probably Low	Probably Low	Low	Probably Low	Definitely Low
Olsen 2019	SALT 90	Probably Low	Probably Low	Definitely Low	Definitely Low	Probably Low	Probably Low	Probably Low	Low	Probably Low	Definitely Low
Olsen 2019	Treatment-related adverse event	Probably Low	Probably Low	Definitely Low	Definitely Low	Probably Low	Probably Low	Probably Low	Low	Probably Low	Definitely Low
Olsen 2019	Severe adverse event	Probably Low	Probably Low	Definitely Low	Definitely Low	Probably Low	Probably Low	Probably Low	Low	Probably Low	Definitely Low

© 2023 Liu M et al. *JAMA Network Open*

Mikhaylov 2022	SALT 50	Definitely Low	Definitely Low	Definitely Low	Definitely Low	Definitely Low	Definitely Low	Definitely Low	High	Low	Definitely Low
Mikhaylov 2022	Treatment-related adverse event	Definitely Low	Definitely Low	Definitely Low	Definitely Low	Definitely Low	Definitely Low	Definitely Low	High	Low	Definitely Low
Mikhaylov 2022	Severe adverse event	Definitely Low	Definitely Low	Definitely Low	Definitely Low	Definitely Low	Definitely Low	Definitely Low	High	Low	Definitely Low
Mikhaylov 2022	Change from baseline of SALT score	Definitely Low	Definitely Low	Definitely Low	Definitely Low	Definitely Low	Definitely Low	Definitely Low	High	Low	Definitely Low
King 2022a	SALT 50	Probably Low	Probably Low	Definitely Low	Definitely Low	Probably Low	Probably Low	Probably Low	Low	Probably Low	Definitely Low
King 2022a	SALT score ≤20	Probably Low	Probably Low	Definitely Low	Definitely Low	Probably Low	Probably Low	Probably Low	Low	Probably Low	Definitely Low
King 2022a	Treatment-related adverse event	Probably Low	Probably Low	Definitely Low	Definitely Low	Probably Low	Probably Low	Probably Low	Low	Probably Low	Definitely Low
King 2022a	Severe adverse event	Probably Low	Probably Low	Definitely Low	Definitely Low	Probably Low	Probably Low	Probably Low	Low	Probably Low	Definitely Low
King 2022a	Discontinuation due to Adverse Event	Probably Low	Probably Low	Definitely Low	Definitely Low	Probably Low	Probably Low	Probably Low	Low	Probably Low	Definitely Low
King 2021b	SALT 30	Definitely Low	Definitely Low	Definitely Low	Definitely Low	Probably Low	Probably Low	Probably Low	Low	Definitely Low	Definitely Low
King 2021b	SALT 50	Definitely Low	Definitely Low	Definitely Low	Definitely Low	Probably Low	Probably Low	Probably Low	Low	Definitely Low	Definitely Low
King 2021b	SALT 90	Definitely Low	Definitely Low	Definitely Low	Definitely Low	Probably Low	Probably Low	Probably Low	Low	Definitely Low	Definitely Low
King 2021b	SALT score ≤20	Definitely Low	Definitely Low	Definitely Low	Definitely Low	Probably Low	Probably Low	Probably Low	Low	Definitely Low	Definitely Low

© 2023 Liu M et al. *JAMA Network Open*

King 2021b	SALT score ≤10	Definitely Low	Definitely Low	Definitely Low	Definitely Low	Probably Low	Probably Low	Probably Low	Low	Definitely Low	Definitely Low
King 2021b	Treatment-related adverse event	Definitely Low	Definitely Low	Definitely Low	Definitely Low	Probably Low	Probably Low	Probably Low	Low	Definitely Low	Definitely Low
King 2021b	Severe adverse event	Definitely Low	Definitely Low	Definitely Low	Definitely Low	Probably Low	Probably Low	Probably Low	Low	Definitely Low	Definitely Low
King 2021b	Change from baseline of SALT score	Definitely Low	Definitely Low	Definitely Low	Definitely Low	Probably Low	Probably Low	Probably Low	Low	Definitely Low	Definitely Low
King 2022b BRAVE-AA1	SALT 90	Definitely Low	Definitely Low	Definitely Low	Definitely Low	Probably Low	Probably Low	Probably Low	Low	Probably Low	Definitely Low
King 2022b BRAVE-AA1	SALT score ≤20	Definitely Low	Definitely Low	Definitely Low	Definitely Low	Probably Low	Probably Low	Probably Low	Low	Probably Low	Definitely Low
King 2022b BRAVE-AA1	SALT score ≤10	Definitely Low	Definitely Low	Definitely Low	Definitely Low	Probably Low	Probably Low	Probably Low	Low	Probably Low	Definitely Low
King 2022b BRAVE-AA1	Treatment-related adverse event	Definitely Low	Definitely Low	Definitely Low	Definitely Low	Probably Low	Probably Low	Probably Low	Low	Probably Low	Definitely Low
King 2022b BRAVE-AA1	Severe adverse event	Definitely Low	Definitely Low	Definitely Low	Definitely Low	Probably Low	Probably Low	Probably Low	Low	Probably Low	Definitely Low
King 2022b BRAVE-AA1	Discontinuation due to Adverse Event	Definitely Low	Definitely Low	Definitely Low	Definitely Low	Probably Low	Probably Low	Probably Low	Low	Probably Low	Definitely Low

© 2023 Liu M et al. *JAMA Network Open*

King 2022b BRAVE-AA1	Change from baseline of SALT score	Definitely Low	Definitely Low	Definitely Low	Definitely Low	Probably Low	Probably Low	Probably Low	Low	Probably Low	Definitely Low
King 2022b BRAVE-AA2	SALT 90	Definitely Low	Definitely Low	Definitely Low	Definitely Low	Probably Low	Probably Low	Probably Low	Probable Low	Probably Low	Definitely Low
King 2022b BRAVE-AA2	SALT score ≤20	Definitely Low	Definitely Low	Definitely Low	Definitely Low	Probably Low	Probably Low	Probably Low	Probable Low	Probably Low	Definitely Low
King 2022b BRAVE-AA2	SALT score ≤10	Definitely Low	Definitely Low	Definitely Low	Definitely Low	Probably Low	Probably Low	Probably Low	Probable Low	Probably Low	Definitely Low
King 2022b BRAVE-AA2	Treatment-related adverse event	Definitely Low	Definitely Low	Definitely Low	Definitely Low	Probably Low	Probably Low	Probably Low	Probable Low	Probably Low	Definitely Low
King 2022b BRAVE-AA2	Severe adverse event	Definitely Low	Definitely Low	Definitely Low	Definitely Low	Probably Low	Probably Low	Probably Low	Probable Low	Probably Low	Definitely Low
King 2022b BRAVE-AA2	Discontinuation due to Adverse Event	Definitely Low	Definitely Low	Definitely Low	Definitely Low	Probably Low	Probably Low	Probably Low	Probable Low	Probably Low	Definitely Low
King 2022b BRAVE-AA2	Change from baseline of SALT score	Definitely Low	Definitely Low	Definitely Low	Definitely Low	Probably Low	Probably Low	Probably Low	Probable Low	Probably Low	Definitely Low

© 2023 Liu M et al. *JAMA Network Open*

eAppendix 2: Detail of Subgroup analysis

Effects of JAK inhibitors

Change from baseline of SALT scores

Subgroup analysis by route of administration, the results showed that oral JAK inhibitors lowered more SALT scores from baseline compared to placebo (MD = - 36.80, 95%CI: -39.57 to -34.02, eFigure 1), and there was no different between external JAK inhibitors and placebo (MD = - 0.40, 95%CI: -11.30 to 10.50; eFigure 1). Applying ICEMAN criteria, we judged the credibility as moderate (eTable 3). Subgroup analysis by different drugs, the results showed that “ritlecitinib” (MD = - 31.10, 95%CI: -37.21 to -24.99; eFigure 2), “brepocitinib” (MD = - 49.20, 95%CI: -55.42 to -49.28; eFigure 2) and “baricitinib” (MD = - 36.28, 95%CI: -39.25 to -33.31; eFigure 2) lowered more SALT scores from baseline compared to placebo. There was no different between “delgocitinib” and placebo (MD= - 0.40, 95%CI: -11.30 to 10.50; eFigure 2). Applying ICEMAN criteria, we judged the credibility as low (eTable 3).

SALT 50

Subgroup analysis by route of administration, the results showed that oral JAK inhibitors enabled more participants achieved a 50% improvement in SALT score from baseline compared to placebo (OR = 12.89, 95%CI: 5.86 to 28.35; eFigure 3), and no different between external JAK inhibitors and placebo (OR = 0.99, 95%CI: 0.33 to 2.95; eFigure 3). Applying ICEMAN criteria, we judged the credibility as moderate (eTable 3). Subgroup analysis by different drugs, the results showed that “ritlecitinib” (OR = 20.49, 95%CI: 3.66 to 114.85; eFigure 4), “brepocitinib” (OR = 32.32, 95%CI: 5.78 to 180.77;

© 2023 Liu M et al. *JAMA Network Open*

eFigure 4) and “baricitinib” (OR = 10.60, 95%CI: 4.34 to 25.89; eFigure 4) enabled more participants achieved a 50% improvement in SALT score from baseline compared to placebo. It is no different between “ruxolitinib” (OR = 1.00, 95%CI: 0.28 to 3.57; eFigure 4) and placebo, and no different between “delgocitinib” (OR = 0.95, 95%CI: 0.11 to 8.21; eFigure 4) and placebo. Applying ICEMAN criteria, we judged the credibility as low (eTable 3).

SALT 90

Subgroup analysis by route of administration, the results showed that oral JAK inhibitors enabled more participants achieved a 90% improvement in SALT score from baseline compared to placebo (OR = 9.56, 95%CI: 4.23 to 21.57; eFigure 5), and no different between external JAK inhibitors and placebo (OR = 5.27, 95%CI: 0.24 to 113.35; eFigure 5). Applying ICEMAN criteria, we judged the credibility as moderate (eTable 3). Subgroup analysis by different drugs, the results showed that “ritlecitinib” (OR = 32.53, 95%CI: 1.86 to 567.78; eFigure 6), “brepocitinib” (OR = 45.31, 95%CI: 2.62 to 784.28; eFigure 6) and “baricitinib” (OR = 8.16, 95%CI: 3.83 to 17.39; eFigure 6) enabled more participants achieved a 90% improvement in SALT score from baseline compared to placebo. It is no different between “ruxolitinib” (OR = 5.27, 95%CI: 0.24 to 113.35; eFigure 6) and placebo. Applying ICEMAN criteria, we judged the credibility as low (eTable 3).

SALT score ≤ 20

Subgroup analysis by different drugs, the results showed that “CTP-543” (OR = 4.79, 95%CI: 1.49 to 15.42; eFigure 7) and “baricitinib” (OR = 7.90, 95%CI: 4.99 to 12.50;

© 2023 Liu M et al. *JAMA Network Open*

eFigure 7) enables more percentage of patients to achieve SALT score ≤ 20 over treatment period compared to placebo. Applying ICEMAN criteria, we judged the credibility as low (eTable 3).

Adverse events of JAK inhibitors

Treatment-related adverse event

Subgroup analysis by route of administration, the results showed that oral JAK inhibitors (RR = 1.07, 95%CI: 0.51 to 2.26; eFigure 8) and external JAK inhibitors (RR = 1.10, 95%CI: 1.00 to 1.21; eFigure 8) all may not cause more treatment-related adverse events compared with placebo. Applying ICEMAN criteria, we judged the credibility as moderate (eTable 3). Subgroup analysis by different drugs, the results showed that "ruxolitinib" (OR = 1.00, 95%CI: 0.40 to 2.49; eFigure 9), "delgocitinib" (OR = 1.23, 95%CI: 0.33 to 4.57; eFigure 9), "CTP-543" (OR = 1.22, 95%CI: 0.82 to 1.81; eFigure 9) and "baricitinib" (OR = 1.09, 95%CI: 0.99 to 1.21; eFigure 9) all may not cause more treatment-related adverse events compared with placebo. Applying ICEMAN criteria, we judged the credibility as low (eTable 3).

Severe adverse event

Subgroup analysis by route of administration, the results showed that oral JAK inhibitors (RR = 0.70, 95%CI: 0.32 to 1.53; eFigure 10) and external JAK inhibitors (RR = 3.00, 95%CI: 0.13 to 71.43; eFigure 10) all may not cause severe adverse events compared with placebo. Applying ICEMAN criteria, we judged the credibility as moderate (eTable 3). Subgroup analysis by different drugs, the results showed that "ritlecitinib" (OR = 0.20, 95%CI: 0.01 to 3.97; eFigure 11), "brepocitinib" (OR = 0.20,

© 2023 Liu M et al. *JAMA Network Open*

95%CI: 0.01 to 4.06; eFigure 11), “ruxolitinib” (OR = 3.00, 95%CI: 0.13 to 71.43; eFigure 11), “CTP-543” (OR = 0.42, 95%CI: 0.08 to 2.35; eFigure 11) and “baricitinib” (OR = 0.92, 95%CI: 0.36 to 2.36; eFigure 11) all may not cause more treatment-related adverse events compared with placebo. Applying ICEMAN criteria, we judged the credibility as low (eTable 3).

Discontinuation due to Adverse Event

Subgroup analysis by route of administration, the results showed that oral JAK inhibitors (RR = 0.89, 95%CI: 0.44 to 1.78; eFigure 12) and external JAK inhibitors (RR = 0.33, 95%CI: 0.01 to 7.94; eFigure 12) all may not cause severe adverse events compared with placebo. Applying ICEMAN criteria, we judged the credibility as moderate (eTable 3). Subgroup analysis by different drugs, the results showed that “ritlecitinib” (OR = 1.63, 95%CI: 0.22 to 11.87; eFigure 13), “brepocitinib” (OR = 1.67, 95%CI: 0.23 to 12.12; eFigure 13), “ruxolitinib” (OR = 0.33, 95%CI: 0.01 to 7.94; eFigure 13), “CTP-543” (OR = 0.30, 95%CI: 0.06 to 1.47; eFigure 13) and “baricitinib” (OR = 1.08, 95%CI: 0.46 to 2.56; eFigure 13) all may not cause more treatment-related adverse events compared with placebo. Applying ICEMAN criteria, we judged the credibility as low (eTable 3).

© 2023 Liu M et al. *JAMA Network Open*

eTable 3. Credibility assessment of subgroup analysis

eTable 3.1 Credibility assessment of route of administration for Change from baseline of SALT scores

1: Is the analysis of effect modification based on comparison within rather than between trials?

[ ] Completely between	[ ] Mostly between or unclear	[ ] Mostly within	[X] Completely within
Subgroup analysis or meta-regression comparing overall effects of each individual trial. This is typical for aggregate data meta-analysis.	*Subgroup analysis or meta-regression with most information coming from overall effects, but some trials providing within-trial subgroup information*	*Most trials providing within-trial subgroup information; or individual participant data analysis that combines within and between trial information*	*All trials providing within-trial subgroup information or individual participant data; and the analysis separates within from between trial information, e.g., meta-analysis of interactions*

Comment: Five trials provided within subgroups used for analysis.

2: For within-trial comparisons, is the effect modification similar from trial to trial? [ ] Not applicable: no or one within-RCT comparison

[ ] Definitely not similar	[ ] Probably not similar or unclear	[ ] Mostly similar	[X] Definitely similar
Effect modification reported for two or more trials and clearly different directions	*Effect modification not reported for individual trials or too imprecise to tell*	*Effect modification reported for two or more trials, mostly similar in direction, but considerable differences in magnitude*	*Effect modification reported for two or more trials, similar in direction, only some differences in magnitude*

Comment: The MD (i.e., the within-trial measure of effect modification) is always in the same direction.

3: For between-trial comparisons, is the number of trials large? [ ] Not applicable: no between RCT comparison

[ ] Very small	[X] Rather small or unclear	[ ] Rather large	[ ] Large
1 or 2 or in smallest subgroup; 5 or less in continuous meta-regression	*3-4 in smallest subgroup; 6-10 in continuous meta-regression*	*5-9 in smallest subgroup; 11 to 15 in continuous meta-regression*	*10 or more in smallest subgroup; more than 15 in continuous meta-regression*

Comment: Two RTCs is a rather larger number and two RCTs is a rather small number.

4: Was the direction of effect modification correctly hypothesised a priori?

[ ] Definitely no	[X] Probably no or unclear	[ ] Probably yes	[ ] Definitely yes
Clearly post-hoc or results inconsistent with hypothesised direction biologically very implausible	*Vague hypothesis or hypothesised or direction unclear*	*No prior protocol available but unequivocal statement of a priori hypothesis with correct direction of effect modification*	*Prior protocol available and includes correct specification of direction of effect modification, e.g., based on a biologic rationale*

Comment: No information.

5: Does a test for interaction suggest that chance is an unlikely explanation of the apparent effect modification? (consider irrespective of number of effect modifiers)

[ ] Chance a very likely explanation	[ ] Chance a likely explanation or unclear	[ ] Chance may not explain	[X] Chance an unlikely explanation
Interaction or meta-regression p-value >0.05	*Interaction or meta-regression p-value ≤0.05 and >0.01, or no test of*	*Interaction or meta-regression p-value ≤0.01 and >0.005*	*Interaction or meta-regression p-value ≤0.005*

© 2023 Liu M et al. *JAMA Network Open*

	interaction reported and not computable		

Comment: The interaction P-value = 0.01.

6: Did the authors test only a small number of effect modifiers or consider the number in their statistical analysis?

[ ] Definitely no	[X] Probably no or unclear	[ ] Probably yes	[ ] Definitely yes
Explicitly exploratory analysis or large number of effect modifiers tested (e.g., greater than 10) and multiplicity not considered in analysis	*No mention of number or 4-10 effect modifiers tested and number not considered in analysis*	*No protocol available but unequivocal statement of 3 or fewer effect modifiers tested*	*Protocol available and 3 or fewer effect modifiers tested or number considered in analysis*

Comment: Two effect modifiers were tested in this review.

7: Did the authors use a random effects model? [ ] Not applicable

[ ] Definitely no	[ ] Probably no or unclear	[ ] Probably yes	[X] Definitely yes
Fixed (or common) effect or fixed effects model explicitly stated	*Probably fixed effect(s) model*	*Probably random (or mixed) effects*	*Random (or mixed) effects explicitly stated*

Comment: Yes, random effects model for combining the interaction estimates.

8: If the effect modifier is a continuous variable, were arbitrary cut points avoided? [ ] not applicable: not continuous

[ ] Definitely no	[ ] Probably no or unclear	[**X**] Probably yes	[X] Definitely yes
Analysis based on exploratory cut point(s), e.g., picking cut point associated with highest interaction p-value	*Analysis based on cut point(s) of unclear origin*	*Analysis based on pre-specified cut point(s), e.g., suggested by prior RCT*	*Analysis based on the full continuum, e.g., assuming a linear or logarithmic relationship*

Comment: We analysis this outcome as continuous variable.

9 Optional: Are there any additional considerations that may increase or decrease credibility? (manual section 3.9) [X] not applicable

	[ ] Yes, probably decrease	[ ] Yes, probably increase
	Biologically implausible<br>Expect similar severe critical<br>Opposite effects unlikely	

Comment:

10: How would you rate the overall credibility of the proposed effect modification?

The overall rating should be driven by the items that decrease credibility. The following provides a sensible strategy:

- All responses definitely or probably decrease credibility or unclear → very low
- Two or more responses definitely decrease credibility → maximum usually low even if all other responses satisfy credibility criteria
- One response definitely decreases credibility → maximum usually moderate even if all other responses satisfy credibility criteria
- Two responses probably decrease credibility → maximum usually moderate even if all other responses satisfy credibility criteria
- No response options definitely or probably decrease credibility → high very likely

Place a mark on the continuous line (or type "x" in editable version)

X

© 2023 Liu M et al. *JAMA Network Open*

Very low credibility	Low credibility	Moderate credibility	High credibility
Very likely no effect modification<br>Use overall effect for each subgroup	Likely no effect modification<br>Use overall effect for each subgroup but note remaining uncertainty	Likely effect modification<br>Use separate effects for each subgroup but note remaining uncertainty	Very likely effect modification<br>Use separate effects for each subgroup

Comment:

© 2023 Liu M et al. *JAMA Network Open*

eTable 3.2 Credibility assessment of different drugs for Change from baseline of SALT scores

1: Is the analysis of effect modification based on comparison within rather than between trials?

[ ] Completely between	[ ] Mostly between or unclear	[ ] Mostly within	[X] Completely within
Subgroup analysis or meta-regression comparing overall effects of each individual trial. This is typical for aggregate data meta-analysis.	*Subgroup analysis or meta-regression with most information coming from overall effects, but some trials providing within-trial subgroup information*	*Most trials providing within-trial subgroup information; or individual participant data analysis that combines within and between trial information*	*All trials providing within-trial subgroup information or individual participant data; and the analysis separates within from between trial information, e.g., meta-analysis of interactions*

Comment: Five trials provided within subgroups used for analysis.

2: For within-trial comparisons, is the effect modification similar from trial to trial? [ ] Not applicable: no or one within-RCT comparison

[ ] Definitely not similar	[ ] Probably not similar or unclear	[ ] Mostly similar	[X] Definitely similar
Effect modification reported for two or more trials and clearly different directions	*Effect modification not reported for individual trials or too imprecise to tell*	*Effect modification reported for two or more trials, mostly similar in direction, but considerable differences in magnitude*	*Effect modification reported for two or more trials, similar in direction, only some differences in magnitude*

Comment: The ratio of OR (i.e., the within-trial measure of effect modification) is always in the same direction.

3: For between-trial comparisons, is the number of trials large? [ ] Not applicable: no between RCT comparison

[ ] Very small	[X] Rather small or unclear	[ ] Rather large	[ ] Large
1 or 2 or in smallest subgroup; 5 or less in continuous meta-regression	*3-4 in smallest subgroup; 6-10 in continuous meta-regression*	*5-9 in smallest subgroup; 11 to 15 in continuous meta-regression*	*10 or more in smallest subgroup; more than 15 in continuous meta-regression*

Comment: Three RTCs is a rather larger number and two RCTs is a rather small number.

4: Was the direction of effect modification correctly hypothesised a priori?

[ ] Definitely no	[X] Probably no or unclear	[ ] Probably yes	[ ] Definitely yes
Clearly post-hoc or results inconsistent with hypothesised direction biologically very implausible	*Vague hypothesis or hypothesised or direction unclear*	*No prior protocol available but unequivocal statement of a priori hypothesis with correct direction of effect modification*	*Prior protocol available and includes correct specification of direction of effect modification, e.g., based on a biologic rationale*

Comment: No information.

5: Does a test for interaction suggest that chance is an unlikely explanation of the apparent effect modification? (consider irrespective of number of effect modifiers)

[ ] Chance a very likely explanation	[ ] Chance a likely explanation or unclear	[ ] Chance may not explain	[X]Chance an unlikely explanation

© 2023 Liu M et al. *JAMA Network Open*

Interaction or meta-regression p-value >0.05	*Interaction or meta-regression p-value ≤0.05 and >0.01, or no test of interaction reported and not computable*	*Interaction or meta-regression p-value ≤0.01 and >0.005*	*Interaction or meta-regression p-value ≤0.005*

Comment: The interaction P-value < 0.01.

6: Did the authors test only a small number of effect modifiers or consider the number in their statistical analysis?

[ ] Definitely no	[X] Probably no or unclear	[ ] Probably yes	[ ] Definitely yes
Explicitly exploratory analysis or large number of effect modifiers tested (e.g., greater than 10) and multiplicity not considered in analysis	*No mention of number or 4-10 effect modifiers tested and number not considered in analysis*	*No protocol available but unequivocal statement of 3 or fewer effect modifiers tested*	*Protocol available and 3 or fewer effect modifiers tested or number considered in analysis*

Comment: Two effect modifiers were tested in this review.

7: Did the authors use a random effects model? [ ] Not applicable

[ ] Definitely no	[ ] Probably no or unclear	[ ] Probably yes	[X] Definitely yes
Fixed (or common) effect or fixed effects model explicitly stated	*Probably fixed effect(s) model*	*Probably random (or mixed) effects*	*Random (or mixed) effects explicitly stated*

Comment: Yes, random effects model for combining the interaction estimates.

8: If the effect modifier is a continuous variable, were arbitrary cut points avoided? [ ] not applicable: not continuous

[ ] Definitely no	[ ] Probably no or unclear	[ ] Probably yes	[X] Definitely yes
Analysis based on exploratory cut point(s), e.g., picking cut point associated with highest interaction p-value	*Analysis based on cut point(s) of unclear origin*	*Analysis based on pre-specified cut point(s), e.g., suggested by prior RCT*	*Analysis based on the full continuum, e.g., assuming a linear or logarithmic relationship*

Comment: We analysis this outcome as continuous variable.

9 Optional: Are there any additional considerations that may increase or decrease credibility? (manual section 3.9) [X] not applicable

	[ ] Yes, probably decrease<br>Biologically implausible<br>Expect similar severe critical<br>Opposite effects unlikely	[ ] Yes, probably increase

Comment:

10: How would you rate the overall credibility of the proposed effect modification?

The overall rating should be driven by the items that decrease credibility. The following provides a sensible strategy:

- All responses definitely or probably decrease credibility or unclear → very low
- Two or more responses definitely decrease credibility → maximum usually low even if all other responses satisfy credibility criteria
- One response definitely decreases credibility → maximum usually moderate even if all other responses satisfy credibility criteria
- Two responses probably decrease credibility → maximum usually moderate even if all other responses satisfy credibility criteria
- No response options definitely or probably decrease credibility → high very likely

Place a mark on the continuous line (or type “x” in editable version)

© 2023 Liu M et al. *JAMA Network Open*

X

Very low credibility	Low credibility	Moderate credibility	High credibility
Very likely no effect modification<br>Use overall effect for each subgroup	Likely no effect modification<br>Use overall effect for each subgroup but note remaining uncertainty	Likely effect modification<br>Use separate effects for each subgroup but note remaining uncertainty	Very likely effect modification<br>Use separate effects for each subgroup

Comment:

© 2023 Liu M et al. *JAMA Network Open*

eTable 3.3 Credibility assessment of route of administration for SALT 50

1: Is the analysis of effect modification based on comparison within rather than between trials?

[ ] Completely between	[ ] Mostly between or unclear	[ ] Mostly within	[X] Completely within
Subgroup analysis or meta-regression comparing overall effects of each individual trial. This is typical for aggregate data meta-analysis.	*Subgroup analysis or meta-regression with most information coming from overall effects, but some trials providing within-trial subgroup information*	*Most trials providing within-trial subgroup information; or individual participant data analysis that combines within and between trial information*	*All trials providing within-trial subgroup information or individual participant data; and the analysis separates within from between trial information, e.g., meta-analysis of interactions*

Comment: Five trials provided within subgroups used for analysis.

2: For within-trial comparisons, is the effect modification similar from trial to trial? [ ] Not applicable: no or one within-RCT comparison

[ ] Definitely not similar	[ ] Probably not similar or unclear	[ ] Mostly similar	[X] Definitely similar
Effect modification reported for two or more trials and clearly different directions	*Effect modification not reported for individual trials or too imprecise to tell*	*Effect modification reported for two or more trials, mostly similar in direction, but considerable differences in magnitude*	*Effect modification reported for two or more trials, similar in direction, only some differences in magnitude*

Comment: The OR (i.e., the within-trial measure of effect modification) is always in the same direction.

3: For between-trial comparisons, is the number of trials large? [ ] Not applicable: no between RCT comparison

[ ] Very small	[X] Rather small or unclear	[ ] Rather large	[ ] Large
1 or 2 or in smallest subgroup; 5 or less in continuous meta-regression	*3-4 in smallest subgroup; 6-10 in continuous meta-regression*	*5-9 in smallest subgroup; 11 to 15 in continuous meta-regression*	*10 or more in smallest subgroup; more than 15 in continuous meta-regression*

Comment: Two RTCs is a rather larger number and two RCTs is a rather small number.

4: Was the direction of effect modification correctly hypothesised a priori?

[X] Definitely no	[X] Probably no or unclear	[ ] Probably yes	[ ] Definitely yes
Clearly post-hoc or results inconsistent with hypothesised direction biologically very implausible	*Vague hypothesis or hypothesised or direction unclear*	*No prior protocol available but unequivocal statement of a priori hypothesis with correct direction of effect modification*	*Prior protocol available and includes correct specification of direction of effect modification, e.g., based on a biologic rationale*

Comment: No information.

5: Does a test for interaction suggest that chance is an unlikely explanation of the apparent effect modification? (consider irrespective of number of effect modifiers)

[ ] Chance a very likely explanation	[ ] Chance a likely explanation or	[ ] Chance may not explain	[X] Chance an unlikely explanation

© 2023 Liu M et al. *JAMA Network Open*

	unclear		
Interaction or meta-regression p-value >0.05	*Interaction or meta-regression p-value ≤0.05 and >0.01, or no test of interaction reported and not computable*	*Interaction or meta-regression p-value ≤0.01 and >0.005*	*Interaction or meta-regression p-value ≤0.005*

Comment: The interaction P-value < 0.01.

6: Did the authors test only a small number of effect modifiers or consider the number in their statistical analysis?

[ ] Definitely no	[X] Probably no or unclear	[ ] Probably yes	[ ] Definitely yes
Explicitly exploratory analysis or large number of effect modifiers tested (e.g., greater than 10) and multiplicity not considered in analysis	*No mention of number or 4-10 effect modifiers tested and number not considered in analysis*	*No protocol available but unequivocal statement of 3 or fewer effect modifiers tested*	*Protocol available and 3 or fewer effect modifiers tested or number considered in analysis*

Comment: Two effect modifiers were tested in this review.

7: Did the authors use a random effects model? [ ] Not applicable

[ ] Definitely no	[ ] Probably no or unclear	[ ] Probably yes	[X] Definitely yes
Fixed (or common) effect or fixed effects model explicitly stated	*Probably fixed effect(s) model*	*Probably random (or mixed) effects*	*Random (or mixed) effects explicitly stated*

Comment: Yes, random effects model for combining the interaction estimates.

8: If the effect modifier is a continuous variable, were arbitrary cut points avoided? [X]not applicable: not continuous

[ ] Definitely no	[ ] Probably no or unclear	[ ] Probably yes	[ ] Definitely yes
Analysis based on exploratory cut point(s), e.g., picking cut point associated with highest interaction p-value	*Analysis based on cut point(s) of unclear origin*	*Analysis based on pre-specified cut point(s), e.g., suggested by prior RCT*	*Analysis based on the full continuum, e.g., assuming a linear or logarithmic relationship*

Comment:

9 Optional: Are there any additional considerations that may increase or decrease credibility? (manual section 3.9) [X] not applicable

	[ ] Yes, probably decrease<br>Biologically implausible<br>Expect similar severe critical<br>Opposite effects unlikely	[ ] Yes, probably increase	

Comment:

© 2023 Liu M et al. *JAMA Network Open*

10: How would you rate the overall credibility of the proposed effect modification?

The overall rating should be driven by the items that decrease credibility. The following provides a sensible strategy:

- All responses definitely or probably decrease credibility or unclear → very low
- Two or more responses definitely decrease credibility → maximum usually low even if all other responses satisfy credibility criteria
- One response definitely decreases credibility → maximum usually moderate even if all other responses satisfy credibility criteria
- Two responses probably decrease credibility → maximum usually moderate even if all other responses satisfy credibility criteria
- No response options definitely or probably decrease credibility → high very likely

Place a mark on the continuous line (or type “x” in editable version)

x

**Very low credibility**	**Low credibility**	**Moderate credibility**	**High credibility**
Very likely no effect modification<br>Use overall effect for each subgroup	Likely no effect modification<br>Use overall effect for each subgroup but note remaining uncertainty	Likely effect modification<br>Use separate effects for each subgroup but note remaining uncertainty	Very likely effect modification<br>Use separate effects for each subgroup

Comment:

© 2023 Liu M et al. *JAMA Network Open*

eTable 3.4 Credibility assessment of different drugs for SALT 50

1: Is the analysis of effect modification based on comparison within rather than between trials?

[ ] Completely between	[ ] Mostly between or unclear	[ ] Mostly within	[X] Completely within
Subgroup analysis or meta-regression comparing overall effects of each individual trial. This is typical for aggregate data meta-analysis.	*Subgroup analysis or meta-regression with most information coming from overall effects, but some trials providing within-trial subgroup information*	*Most trials providing within-trial subgroup information; or individual participant data analysis that combines within and between trial information*	*All trials providing within-trial subgroup information or individual participant data; and the analysis separates within from between trial information, e.g., meta-analysis of interactions*

Comment: Five trials provided within subgroups used for analysis.

2: For within-trial comparisons, is the effect modification similar from trial to trial? [ ] Not applicable: no or one within-RCT comparison

[ ] Definitely not similar	[ ] Probably not similar or unclear	[ ] Mostly similar	[X] Definitely similar
Effect modification reported for two or more trials and clearly different directions	*Effect modification not reported for individual trials or too imprecise to tell*	*Effect modification reported for two or more trials, mostly similar in direction, but considerable differences in magnitude*	*Effect modification reported for two or more trials, similar in direction, only some differences in magnitude*

Comment: The ratio of OR (i.e., the within-trial measure of effect modification) is always in the same direction.

3: For between-trial comparisons, is the number of trials large? [ ] Not applicable: no between RCT comparison

[ ] Very small	[X] Rather small or unclear	[ ] Rather large	[ ] Large
1 or 2 or in smallest subgroup; 5 or less in continuous meta-regression	*3-4 in smallest subgroup; 6-10 in continuous meta-regression*	*5-9 in smallest subgroup; 11 to 15 in continuous meta-regression*	*10 or more in smallest subgroup; more than 15 in continuous meta-regression*

Comment: Three RTCs is a rather larger number and two RCTs is a rather small number.

© 2023 Liu M et al. *JAMA Network Open*

4: Was the direction of effect modification correctly hypothesised a priori?

[ ] Definitely no	[X] Probably no or unclear	[ ] Probably yes	[ ] Definitely yes
Clearly post-hoc or results inconsistent with hypothesised direction biologically very implausible	*Vague hypothesis or hypothesised or direction unclear*	*No prior protocol available but unequivocal statement of a priori hypothesis with correct direction of effect modification*	*Prior protocol available and includes correct specification of direction of effect modification, e.g., based on a biologic rationale*

Comment: No information.

5: Does a test for interaction suggest that chance is an unlikely explanation of the apparent effect modification? (consider irrespective of number of effect modifiers)

[ ] Chance a very likely explanation	[ ] Chance a likely explanation or unclear	[ ] Chance may not explain	[X]Chance an unlikely explanation
Interaction or meta-regression p-value >0.05	*Interaction or meta-regression p-value ≤0.05 and >0.01, or no test of interaction reported and not computable*	*Interaction or meta-regression p-value ≤0.01 and >0.005*	*Interaction or meta-regression p-value ≤0.005*

Comment: The interaction P-value < 0.01.

6: Did the authors test only a small number of effect modifiers or consider the number in their statistical analysis?

[ ] Definitely no	[X] Probably no or unclear	[ ] Probably yes	[ ] Definitely yes
Explicitly exploratory analysis or large number of effect modifiers tested (e.g., greater than 10) and multiplicity not considered in analysis	*No mention of number or 4-10 effect modifiers tested and number not considered in analysis*	*No protocol available but unequivocal statement of 3 or fewer effect modifiers tested*	*Protocol available and 3 or fewer effect modifiers tested or number considered in analysis*

Comment: Two effect modifiers were tested in this review.

7: Did the authors use a random effects model? [ ] Not applicable

[ ] Definitely no	[ ] Probably no or unclear	[ ] Probably yes	[X] Definitely yes
Fixed (or common) effect or fixed effects model explicitly stated	*Probably fixed effect(s) model*	*Probably random (or mixed) effects*	*Random (or mixed) effects explicitly stated*

Comment: Yes, random effects model for combining the interaction estimates.

8: If the effect modifier is a continuous variable, were arbitrary cut points avoided? [X] not applicable: not continuous

[ ] Definitely no	[ ] Probably no or unclear	[ ] Probably yes	[ ] Definitely yes
Analysis based on exploratory cut point(s), e.g., picking cut point associated with highest interaction p-value	*Analysis based on cut point(s) of unclear origin*	*Analysis based on pre-specified cut point(s), e.g., suggested by prior RCT*	*Analysis based on the full continuum, e.g., assuming a linear or logarithmic relationship*

Comment:

9 Optional: Are there any additional considerations that may increase or decrease credibility? (manual section 3.9) [X] not applicable

	[ ] Yes, probably decrease<br>Biologically implausible<br>Expect similar severe critical	[ ] Yes, probably increase	

© 2023 Liu M et al. *JAMA Network Open*

Opposite effects unlikely

Comment:

10: How would you rate the overall credibility of the proposed effect modification?

The overall rating should be driven by the items that decrease credibility. The following provides a sensible strategy:

- All responses definitely or probably decrease credibility or unclear → very low
- Two or more responses definitely decrease credibility → maximum usually low even if all other responses satisfy credibility criteria
- One response definitely decreases credibility → maximum usually moderate even if all other responses satisfy credibility criteria
- Two responses probably decrease credibility → maximum usually moderate even if all other responses satisfy credibility criteria
- No response options definitely or probably decrease credibility → high very likely

Place a mark on the continuous line (or type “x” in editable version)

X

**Very low credibility**	**Low credibility**	**Moderate credibility**	**High credibility**
Very likely no effect modification<br>Use overall effect for each subgroup	Likely no effect modification<br>Use overall effect for each subgroup but note remaining uncertainty	Likely effect modification<br>Use separate effects for each subgroup but note remaining uncertainty	Very likely effect modification<br>Use separate effects for each subgroup

Comment:

© 2023 Liu M et al. *JAMA Network Open*

eTable 3.5 Credibility assessment of route of administration for SALT 90

1: Is the analysis of effect modification based on comparison within rather than between trials?

[ ] Completely between	[ ] Mostly between or unclear	[ ] Mostly within	[X] Completely within
Subgroup analysis or meta-regression comparing overall effects of each individual trial. This is typical for aggregate data meta-analysis.	*Subgroup analysis or meta-regression with most information coming from overall effects, but some trials providing within-trial subgroup information*	*Most trials providing within-trial subgroup information; or individual participant data analysis that combines within and between trial information*	*All trials providing within-trial subgroup information or individual participant data; and the analysis separates within from between trial information, e.g., meta-analysis of interactions*

Comment: Five trials provided within subgroups used for analysis.

2: For within-trial comparisons, is the effect modification similar from trial to trial? [ ] Not applicable: no or one within-RCT comparison

[ ] Definitely not similar	[ ] Probably not similar or unclear	[ ] Mostly similar	[X] Definitely similar
Effect modification reported for two or more trials and clearly different directions	*Effect modification not reported for individual trials or too imprecise to tell*	*Effect modification reported for two or more trials, mostly similar in direction, but considerable differences in magnitude*	*Effect modification reported for two or more trials, similar in direction, only some differences in magnitude*

Comment: The OR (i.e., the within-trial measure of effect modification) is always in the same direction.

3: For between-trial comparisons, is the number of trials large? [ ] Not applicable: no between RCT comparison

[ ] Very small	[X] Rather small or unclear	[ ] Rather large	[ ] Large
1 or 2 or in smallest subgroup; 5 or less in continuous meta-regression	*3-4 in smallest subgroup; 6-10 in continuous meta-regression*	*5-9 in smallest subgroup; 11 to 15 in continuous meta-regression*	*10 or more in smallest subgroup; more than 15 in continuous meta-*

© 2023 Liu M et al. *JAMA Network Open*

			regression

Comment: Two RTCs is a rather larger number and two RCTs is a rather small number.

4: Was the direction of effect modification correctly hypothesised a priori?

[**X**] Definitely no	[**X**] Probably no or unclear	[ ] Probably yes	[ ] Definitely yes
Clearly post-hoc or results inconsistent with hypothesised direction biologically very implausible	*Vague hypothesis or hypothesised or direction unclear*	*No prior protocol available but unequivocal statement of a priori hypothesis with correct direction of effect modification*	*Prior protocol available and includes correct specification of direction of effect modification, e.g., based on a biologic rationale*

Comment: No information.

5: Does a test for interaction suggest that chance is an unlikely explanation of the apparent effect modification? (consider irrespective of number of effect modifiers)

[ ] Chance a very likely explanation	[ ] Chance a likely explanation or unclear	[ ] Chance may not explain	[**X**] Chance an unlikely explanation
Interaction or meta-regression p-value >0.05	*Interaction or meta-regression p-value ≤0.05 and >0.01, or no test of interaction reported and not computable*	*Interaction or meta-regression p-value ≤0.01 and >0.005*	*Interaction or meta-regression p-value ≤0.005*

Comment: The interaction P-value < 0.01.

6: Did the authors test only a small number of effect modifiers or consider the number in their statistical analysis?

[ ] Definitely no	[**X**] Probably no or unclear	[ ] Probably yes	[ ] Definitely yes
Explicitly exploratory analysis or large number of effect modifiers tested (e.g., greater than 10) and multiplicity not considered in analysis	*No mention of number or 4-10 effect modifiers tested and number not considered in analysis*	*No protocol available but unequivocal statement of 3 or fewer effect modifiers tested*	*Protocol available and 3 or fewer effect modifiers tested or number considered in analysis*

Comment: Two effect modifiers were tested in this review.

7: Did the authors use a random effects model? [ ] Not applicable

[ ] Definitely no	[ ] Probably no or unclear	[ ] Probably yes	[**X**] Definitely yes
Fixed (or common) effect or fixed effects model explicitly stated	*Probably fixed effect(s) model*	*Probably random (or mixed) effects*	*Random (or mixed) effects explicitly stated*

Comment: Yes, random effects model for combining the interaction estimates.

8: If the effect modifier is a continuous variable, were arbitrary cut points avoided? [**X**]not applicable: not continuous

[ ] Definitely no	[ ] Probably no or unclear	[ ] Probably yes	[ ] Definitely yes
Analysis based on exploratory cut point(s), e.g., picking cut point associated with highest interaction p-value	*Analysis based on cut point(s) of unclear origin*	*Analysis based on pre-specified cut point(s), e.g., suggested by prior RCT*	*Analysis based on the full continuum, e.g., assuming a linear or logarithmic relationship*

Comment:

9 Optional: Are there any additional considerations that may increase or decrease credibility? (manual section 3.9) [**X**] not applicable

	[ ] Yes, probably decrease	[ ] Yes, probably increase	

© 2023 Liu M et al. *JAMA Network Open*

Biologically implausible

Expect similar severe critical

Opposite effects unlikely

Comment:

10: How would you rate the overall credibility of the proposed effect modification?

The overall rating should be driven by the items that decrease credibility. The following provides a sensible strategy:

- All responses definitely or probably decrease credibility or unclear → very low
- Two or more responses definitely decrease credibility → maximum usually low even if all other responses satisfy credibility criteria
- One response definitely decreases credibility → maximum usually moderate even if all other responses satisfy credibility criteria
- Two responses probably decrease credibility → maximum usually moderate even if all other responses satisfy credibility criteria
- No response options definitely or probably decrease credibility → high very likely

Place a mark on the continuous line (or type "x" in editable version)

x

**Very low credibility**	**Low credibility**	**Moderate credibility**	**High credibility**
Very likely no effect modification<br>Use overall effect for each subgroup	Likely no effect modification<br>Use overall effect for each subgroup but note remaining uncertainty	Likely effect modification<br>Use separate effects for each subgroup but note remaining uncertainty	Very likely effect modification<br>Use separate effects for each subgroup

Comment:

© 2023 Liu M et al. *JAMA Network Open*

eTable 3.6 Credibility assessment of different drugs for SALT 90

1: Is the analysis of effect modification based on comparison within rather than between trials?

[ ] Completely between	[ ] Mostly between or unclear	[ ] Mostly within	[X] Completely within
Subgroup analysis or meta-regression comparing overall effects of each individual trial. This is typical for aggregate data meta-analysis.	*Subgroup analysis or meta-regression with most information coming from overall effects, but some trials providing within-trial subgroup information*	*Most trials providing within-trial subgroup information; or individual participant data analysis that combines within and between trial information*	*All trials providing within-trial subgroup information or individual participant data; and the analysis separates within from between trial information, e.g., meta-analysis of interactions*

Comment: Five trials provided within subgroups used for analysis.

2: For within-trial comparisons, is the effect modification similar from trial to trial? [ ] Not applicable: no or one within-RCT comparison

[ ] Definitely not similar	[ ] Probably not similar or unclear	[ ] Mostly similar	[X] Definitely similar
Effect modification reported for two or more trials and clearly different directions	*Effect modification not reported for individual trials or too imprecise to tell*	*Effect modification reported for two or more trials, mostly similar in direction, but considerable differences in magnitude*	*Effect modification reported for two or more trials, similar in direction, only some differences in magnitude*

Comment: The ratio of OR (i.e., the within-trial measure of effect modification) is always in the same direction.

3: For between-trial comparisons, is the number of trials large? [ ] Not applicable: no between RCT comparison

[ ] Very small	[X] Rather small or unclear	[ ] Rather large	[ ] Large
1 or 2 or in smallest subgroup; 5 or less in continuous meta-regression	*3-4 in smallest subgroup; 6-10 in continuous meta-regression*	*5-9 in smallest subgroup; 11 to 15 in continuous meta-regression*	*10 or more in smallest subgroup; more than 15 in continuous meta-regression*

Comment: Three RTCs is a rather larger number and two RCTs is a rather small number.

4: Was the direction of effect modification correctly hypothesised a priori?

[ ] Definitely no	[X] Probably no or unclear	[ ] Probably yes	[ ] Definitely yes
Clearly post-hoc or results inconsistent with hypothesised direction biologically very implausible	*Vague hypothesis or hypothesised or direction unclear*	*No prior protocol available but unequivocal statement of a priori hypothesis with correct direction of effect modification*	*Prior protocol available and includes correct specification of direction of effect modification, e.g., based on a biologic rationale*

Comment: No information.

5: Does a test for interaction suggest that chance is an unlikely explanation of the apparent effect modification? (consider irrespective of number of effect modifiers)

[ ] Chance a very likely explanation	[ ] Chance a likely explanation or unclear	[ ] Chance may not explain	[X]Chance an unlikely explanation
Interaction or meta-regression p-value >0.05	*Interaction or meta-regression p-value ≤0.05 and >0.01, or no test of interaction reported and not*	*Interaction or meta-regression p-value ≤0.01 and >0.005*	*Interaction or meta-regression p-value ≤0.005*

© 2023 Liu M et al. *JAMA Network Open*

	computable		

Comment: The interaction P-value < 0.01.

6: Did the authors test only a small number of effect modifiers or consider the number in their statistical analysis?

[ ] Definitely no	[X] Probably no or unclear	[ ] Probably yes	[ ] Definitely yes
Explicitly exploratory analysis or large number of effect modifiers tested (e.g., greater than 10) and multiplicity not considered in analysis	*No mention of number or 4-10 effect modifiers tested and number not considered in analysis*	*No protocol available but unequivocal statement of 3 or fewer effect modifiers tested*	*Protocol available and 3 or fewer effect modifiers tested or number considered in analysis*

Comment: Two effect modifiers were tested in this review.

7: Did the authors use a random effects model? [ ] Not applicable

[ ] Definitely no	[ ] Probably no or unclear	[ ] Probably yes	[X] Definitely yes
Fixed (or common) effect or fixed effects model explicitly stated	*Probably fixed effect(s) model*	*Probably random (or mixed) effects*	*Random (or mixed) effects explicitly stated*

Comment: Yes, random effects model for combining the interaction estimates.

8: If the effect modifier is a continuous variable, were arbitrary cut points avoided? [X] not applicable: not continuous

[ ] Definitely no	[ ] Probably no or unclear	[ ] Probably yes	[ ] Definitely yes
Analysis based on exploratory cut point(s), e.g., picking cut point associated with highest interaction p-value	*Analysis based on cut point(s) of unclear origin*	*Analysis based on pre-specified cut point(s), e.g., suggested by prior RCT*	*Analysis based on the full continuum, e.g., assuming a linear or logarithmic relationship*

Comment:

9 Optional: Are there any additional considerations that may increase or decrease credibility? (manual section 3.9) [X] not applicable

	[ ] Yes, probably decrease<br>Biologically implausible<br>Expect similar severe critical<br>Opposite effects unlikely	[ ] Yes, probably increase	

Comment:

10: How would you rate the overall credibility of the proposed effect modification?

The overall rating should be driven by the items that decrease credibility. The following provides a sensible strategy:

- All responses definitely or probably decrease credibility or unclear → very low
- Two or more responses definitely decrease credibility → maximum usually low even if all other responses satisfy credibility criteria
- One response definitely decreases credibility → maximum usually moderate even if all other responses satisfy credibility criteria
- Two responses probably decrease credibility → maximum usually moderate even if all other responses satisfy credibility criteria
- No response options definitely or probably decrease credibility → high very likely

Place a mark on the continuous line (or type "x" in editable version)

X

© 2023 Liu M et al. *JAMA Network Open*

Very low credibility	Low credibility	Moderate credibility	High credibility
Very likely no effect modification<br>Use overall effect for each subgroup	Likely no effect modification<br>Use overall effect for each subgroup but note remaining uncertainty	Likely effect modification<br>Use separate effects for each subgroup but note remaining uncertainty	Very likely effect modification<br>Use separate effects for each subgroup

Comment:

© 2023 Liu M et al. *JAMA Network Open*

eTable 3.7 Credibility assessment of different drugs for SALT score ≤ 20

1: Is the analysis of effect modification based on comparison within rather than between trials?

[ ] Completely between	[ ] Mostly between or unclear	[ ] Mostly within	[X] Completely within
Subgroup analysis or meta-regression comparing overall effects of each individual trial. This is typical for aggregate data meta-analysis.	*Subgroup analysis or meta-regression with most information coming from overall effects, but some trials providing within-trial subgroup information*	*Most trials providing within-trial subgroup information; or individual participant data analysis that combines within and between trial information*	*All trials providing within-trial subgroup information or individual participant data; and the analysis separates within from between trial information, e.g., meta-analysis of interactions*

Comment: Five trials provided within subgroups used for analysis.

2: For within-trial comparisons, is the effect modification similar from trial to trial? [ ] Not applicable: no or one within-RCT comparison

[ ] Definitely not similar	[ ] Probably not similar or unclear	[ ] Mostly similar	[X] Definitely similar
Effect modification reported for two or more trials and clearly different directions	*Effect modification not reported for individual trials or too imprecise to tell*	*Effect modification reported for two or more trials, mostly similar in direction, but considerable differences in magnitude*	*Effect modification reported for two or more trials, similar in direction, only some differences in magnitude*

Comment: The ratio of OR (i.e., the within-trial measure of effect modification) is always in the same direction.

3: For between-trial comparisons, is the number of trials large? [ ] Not applicable: no between RCT comparison

[ ] Very small	[X] Rather small or unclear	[ ] Rather large	[ ] Large
1 or 2 or in smallest subgroup; 5 or less in continuous meta-regression	*3-4 in smallest subgroup; 6-10 in continuous meta-regression*	*5-9 in smallest subgroup; 11 to 15 in continuous meta-regression*	*10 or more in smallest subgroup; more than 15 in continuous meta-regression*

Comment: Three RTCs is a rather larger number and two RCTs is a rather small number.

4: Was the direction of effect modification correctly hypothesised a priori?

[ ] Definitely no	[X] Probably no or unclear	[ ] Probably yes	[ ] Definitely yes
Clearly post-hoc or results inconsistent with hypothesised direction biologically very implausible	*Vague hypothesis or hypothesised or direction unclear*	*No prior protocol available but unequivocal statement of a priori hypothesis with correct direction of effect modification*	*Prior protocol available and includes correct specification of direction of effect modification, e.g., based on a biologic rationale*

Comment: No information.

5: Does a test for interaction suggest that chance is an unlikely explanation of the apparent effect modification? (consider irrespective of number of effect modifiers)

[ ] Chance a very likely explanation	[ ] Chance a likely explanation or unclear	[ ] Chance may not explain	[X]Chance an unlikely explanation
Interaction or meta-regression p-value >0.05	*Interaction or meta-regression p-value ≤0.05 and >0.01, or no test of interaction reported and not*	*Interaction or meta-regression p-value ≤0.01 and >0.005*	*Interaction or meta-regression p-value ≤0.005*

© 2023 Liu M et al. *JAMA Network Open*

	computable		

Comment: The interaction P-value < 0.01.

6: Did the authors test only a small number of effect modifiers or consider the number in their statistical analysis?

[ ] Definitely no	[X] Probably no or unclear	[ ] Probably yes	[ ] Definitely yes
Explicitly exploratory analysis or large number of effect modifiers tested (e.g., greater than 10) and multiplicity not considered in analysis	*No mention of number or 4-10 effect modifiers tested and number not considered in analysis*	*No protocol available but unequivocal statement of 3 or fewer effect modifiers tested*	*Protocol available and 3 or fewer effect modifiers tested or number considered in analysis*

Comment: Two effect modifiers were tested in this review.

7: Did the authors use a random effects model? [ ] Not applicable

[ ] Definitely no	[ ] Probably no or unclear	[ ] Probably yes	[X] Definitely yes
Fixed (or common) effect or fixed effects model explicitly stated	*Probably fixed effect(s) model*	*Probably random (or mixed) effects*	*Random (or mixed) effects explicitly stated*

Comment: Yes, random effects model for combining the interaction estimates.

8: If the effect modifier is a continuous variable, were arbitrary cut points avoided? [X] not applicable: not continuous

[ ] Definitely no	[ ] Probably no or unclear	[ ] Probably yes	[ ] Definitely yes
Analysis based on exploratory cut point(s), e.g., picking cut point associated with highest interaction p-value	*Analysis based on cut point(s) of unclear origin*	*Analysis based on pre-specified cut point(s), e.g., suggested by prior RCT*	*Analysis based on the full continuum, e.g., assuming a linear or logarithmic relationship*

Comment:

9 Optional: Are there any additional considerations that may increase or decrease credibility? (manual section 3.9) [X] not applicable

	[ ] Yes, probably decrease<br>Biologically implausible<br>Expect similar severe critical<br>Opposite effects unlikely	[ ] Yes, probably increase	

Comment:

10: How would you rate the overall credibility of the proposed effect modification?

The overall rating should be driven by the items that decrease credibility. The following provides a sensible strategy:

- All responses definitely or probably decrease credibility or unclear → very low
- Two or more responses definitely decrease credibility → maximum usually low even if all other responses satisfy credibility criteria
- One response definitely decreases credibility → maximum usually moderate even if all other responses satisfy credibility criteria
- Two responses probably decrease credibility → maximum usually moderate even if all other responses satisfy credibility criteria
- No response options definitely or probably decrease credibility → high very likely

Place a mark on the continuous line (or type “x” in editable version)

x

© 2023 Liu M et al. *JAMA Network Open*

Very low credibility	Low credibility	Moderate credibility	High credibility
Very likely no effect modification<br>Use overall effect for each subgroup	Likely no effect modification<br>Use overall effect for each subgroup but note remaining uncertainty	Likely effect modification<br>Use separate effects for each subgroup but note remaining uncertainty	Very likely effect modification<br>Use separate effects for each subgroup

Comment:

© 2023 Liu M et al. *JAMA Network Open*

eTable 3.8 Credibility assessment of route of administration for Treatment-related adverse event

1: Is the analysis of effect modification based on comparison within rather than between trials?

[ ] Completely between	[ ] Mostly between or unclear	[ ] Mostly within	[X] Completely within
Subgroup analysis or meta-regression comparing overall effects of each individual trial. This is typical for aggregate data meta-analysis.	*Subgroup analysis or meta-regression with most information coming from overall effects, but some trials providing within-trial subgroup information*	*Most trials providing within-trial subgroup information; or individual participant data analysis that combines within and between trial information*	*All trials providing within-trial subgroup information or individual participant data; and the analysis separates within from between trial information, e.g., meta-analysis of interactions*

Comment: Five trials provided within subgroups used for analysis.

2: For within-trial comparisons, is the effect modification similar from trial to trial? [ ] Not applicable: no or one within-RCT comparison

[ ] Definitely not similar	[ ] Probably not similar or unclear	[ ] Mostly similar	[X] Definitely similar
Effect modification reported for two or more trials and clearly different directions	*Effect modification not reported for individual trials or too imprecise to tell*	*Effect modification reported for two or more trials, mostly similar in direction, but considerable differences in magnitude*	*Effect modification reported for two or more trials, similar in direction, only some differences in magnitude*

Comment: The OR (i.e., the within-trial measure of effect modification) is always in the same direction.

3: For between-trial comparisons, is the number of trials large? [ ] Not applicable: no between RCT comparison

[ ] Very small	[X] Rather small or unclear	[ ] Rather large	[ ] Large
1 or 2 or in smallest subgroup; 5 or less in continuous meta-regression	*3-4 in smallest subgroup; 6-10 in continuous meta-regression*	*5-9 in smallest subgroup; 11 to 15 in continuous meta-regression*	*10 or more in smallest subgroup; more than 15 in continuous meta-regression*

Comment: Two RTCs is a rather larger number and two RCTs is a rather small number.

4: Was the direction of effect modification correctly hypothesised a priori?

[**X**] Definitely no	[X] Probably no or unclear	[ ] Probably yes	[ ] Definitely yes
Clearly post-hoc or results inconsistent with hypothesised direction biologically very implausible	*Vague hypothesis or hypothesised or direction unclear*	*No prior protocol available but unequivocal statement of a priori hypothesis with correct direction of effect modification*	*Prior protocol available and includes correct specification of direction of effect modification, e.g., based on a biologic rationale*

Comment: No information.

5: Does a test for interaction suggest that chance is an unlikely explanation of the apparent effect modification? (consider irrespective of number of effect modifiers)

[ ] Chance a very likely explanation	[ ] Chance a likely explanation or unclear	[ ] Chance may not explain	[X] Chance an unlikely explanation
Interaction or meta-regression p-value >0.05	*Interaction or meta-regression p-value ≤0.05 and >0.01, or no test of interaction reported and not*	*Interaction or meta-regression p-value ≤0.01 and >0.005*	*Interaction or meta-regression p-value ≤0.005*

© 2023 Liu M et al. *JAMA Network Open*

	computable		

Comment: The interaction P-value < 0.01.

6: Did the authors test only a small number of effect modifiers or consider the number in their statistical analysis?

[ ] Definitely no	[X] Probably no or unclear	[ ] Probably yes	[ ] Definitely yes
Explicitly exploratory analysis or large number of effect modifiers tested (e.g., greater than 10) and multiplicity not considered in analysis	*No mention of number or 4-10 effect modifiers tested and number considered in analysis*	*No protocol available but unequivocal statement of 3 or fewer effect modifiers tested*	*Protocol available and 3 or fewer effect modifiers tested or number considered in analysis*

Comment: Two effect modifiers were tested in this review.

7: Did the authors use a random effects model? [ ] Not applicable

[ ] Definitely no	[ ] Probably no or unclear	[ ] Probably yes	[X] Definitely yes
Fixed (or common) effect or fixed effects model explicitly stated	*Probably fixed effect(s) model*	*Probably random (or mixed) effects*	*Random (or mixed) effects explicitly stated*

Comment: Yes, random effects model for combining the interaction estimates.

8: If the effect modifier is a continuous variable, were arbitrary cut points avoided? [X]not applicable: not continuous

[ ] Definitely no	[ ] Probably no or unclear	[ ] Probably yes	[ ] Definitely yes
Analysis based on exploratory cut point(s), e.g., picking cut point associated with highest interaction p-value	*Analysis based on cut point(s) of unclear origin*	*Analysis based on pre-specified cut point(s), e.g., suggested by prior RCT*	*Analysis based on the full continuum, e.g., assuming a linear or logarithmic relationship*

Comment:

9 Optional: Are there any additional considerations that may increase or decrease credibility? (manual section 3.9) [X] not applicable

	[ ] Yes, probably decrease	[ ] Yes, probably increase	
	Biologically implausible<br>Expect similar severe critical<br>Opposite effects unlikely		

Comment:

10: How would you rate the overall credibility of the proposed effect modification?

The overall rating should be driven by the items that decrease credibility. The following provides a sensible strategy:

- All responses definitely or probably decrease credibility or unclear → very low
- Two or more responses definitely decrease credibility → maximum usually low even if all other responses satisfy credibility criteria
- One response definitely decreases credibility → maximum usually moderate even if all other responses satisfy credibility criteria
- Two responses probably decrease credibility → maximum usually moderate even if all other responses satisfy credibility criteria
- No response options definitely or probably decrease credibility → high very likely

Place a mark on the continuous line (or type "x" in editable version)

x

© 2023 Liu M et al. *JAMA Network Open*

Very low credibility	Low credibility	Moderate credibility	High credibility
Very likely no effect modification<br>Use overall effect for each subgroup	Likely no effect modification<br>Use overall effect for each subgroup but note remaining uncertainty	Likely effect modification<br>Use separate effects for each subgroup but note remaining uncertainty	Very likely effect modification<br>Use separate effects for each subgroup

Comment:

© 2023 Liu M et al. *JAMA Network Open*

eTable 3.9 Credibility assessment of different drugs for Treatment-related adverse event

1: Is the analysis of effect modification based on comparison within rather than between trials?

[ ] Completely between	[ ] Mostly between or unclear	[ ] Mostly within	[X] Completely within
Subgroup analysis or meta-regression comparing overall effects of each individual trial. This is typical for aggregate data meta-analysis.	*Subgroup analysis or meta-regression with most information coming from overall effects, but some trials providing within-trial subgroup information*	*Most trials providing within-trial subgroup information; or individual participant data analysis that combines within and between trial information*	*All trials providing within-trial subgroup information or individual participant data; and the analysis separates within from between trial information, e.g., meta-analysis of interactions*

Comment: Five trials provided within subgroups used for analysis.

2: For within-trial comparisons, is the effect modification similar from trial to trial? [ ] Not applicable: no or one within-RCT comparison

[ ] Definitely not similar	[ ] Probably not similar or unclear	[ ] Mostly similar	[X] Definitely similar
Effect modification reported for two or more trials and clearly different directions	*Effect modification not reported for individual trials or too imprecise to tell*	*Effect modification reported for two or more trials, mostly similar in direction, but considerable differences in magnitude*	*Effect modification reported for two or more trials, similar in direction, only some differences in magnitude*

Comment: The ratio of OR (i.e., the within-trial measure of effect modification) is always in the same direction.

3: For between-trial comparisons, is the number of trials large? [ ] Not applicable: no between RCT comparison

[ ] Very small	[X] Rather small or unclear	[ ] Rather large	[ ] Large
1 or 2 or in smallest subgroup; 5 or less in continuous meta-regression	*3-4 in smallest subgroup; 6-10 in continuous meta-regression*	*5-9 in smallest subgroup; 11 to 15 in continuous meta-regression*	*10 or more in smallest subgroup; more than 15 in continuous meta-regression*

Comment: Three RTCs is a rather larger number and two RCTs is a rather small number.

4: Was the direction of effect modification correctly hypothesised a priori?

[ ] Definitely no	[X] Probably no or unclear	[ ] Probably yes	[ ] Definitely yes
Clearly post-hoc or results inconsistent with hypothesised direction biologically very implausible	*Vague hypothesis or hypothesised or direction unclear*	*No prior protocol available but unequivocal statement of a priori hypothesis with correct direction of effect modification*	*Prior protocol available and includes correct specification of direction of effect modification, e.g., based on a biologic rationale*

Comment: No information.

5: Does a test for interaction suggest that chance is an unlikely explanation of the apparent effect modification? (consider irrespective of number of effect modifiers)

[ ] Chance a very likely explanation	[ ] Chance a likely explanation or unclear	[ ] Chance may not explain	[X]Chance an unlikely explanation
Interaction or meta-regression p-value >0.05	*Interaction or meta-regression p-value ≤0.05 and >0.01, or no test of interaction reported and not*	*Interaction or meta-regression p-value ≤0.01 and >0.005*	*Interaction or meta-regression p-value ≤0.005*

© 2023 Liu M et al. *JAMA Network Open*

	computable		

Comment: The interaction P-value < 0.01.

6: Did the authors test only a small number of effect modifiers or consider the number in their statistical analysis?

[ ] Definitely no	[X] Probably no or unclear	[ ] Probably yes	[ ] Definitely yes
Explicitly exploratory analysis or large number of effect modifiers tested (e.g., greater than 10) and multiplicity not considered in analysis	*No mention of number or 4-10 effect modifiers tested and number not considered in analysis*	*No protocol available but unequivocal statement of 3 or fewer effect modifiers tested*	*Protocol available and 3 or fewer effect modifiers tested or number considered in analysis*

Comment: Two effect modifiers were tested in this review.

7: Did the authors use a random effects model? [ ] Not applicable

[ ] Definitely no	[ ] Probably no or unclear	[ ] Probably yes	[X] Definitely yes
Fixed (or common) effect or fixed effects model explicitly stated	*Probably fixed effect(s) model*	*Probably random (or mixed) effects*	*Random (or mixed) effects explicitly stated*

Comment: Yes, random effects model for combining the interaction estimates.

8: If the effect modifier is a continuous variable, were arbitrary cut points avoided? [X] not applicable: not continuous

[ ] Definitely no	[ ] Probably no or unclear	[ ] Probably yes	[ ] Definitely yes
Analysis based on exploratory cut point(s), e.g., picking cut point associated with highest interaction p-value	*Analysis based on cut point(s) of unclear origin*	*Analysis based on pre-specified cut point(s), e.g., suggested by prior RCT*	*Analysis based on the full continuum, e.g., assuming a linear or logarithmic relationship*

Comment:

9 Optional: Are there any additional considerations that may increase or decrease credibility? (manual section 3.9) [X] not applicable

	[ ] Yes, probably decrease<br>Biologically implausible<br>Expect similar severe critical<br>Opposite effects unlikely	[ ] Yes, probably increase	

Comment:

10: How would you rate the overall credibility of the proposed effect modification?

The overall rating should be driven by the items that decrease credibility. The following provides a sensible strategy:

- All responses definitely or probably decrease credibility or unclear → very low
- Two or more responses definitely decrease credibility → maximum usually low even if all other responses satisfy credibility criteria
- One response definitely decreases credibility → maximum usually moderate even if all other responses satisfy credibility criteria
- Two responses probably decrease credibility → maximum usually moderate even if all other responses satisfy credibility criteria
- No response options definitely or probably decrease credibility → high very likely

Place a mark on the continuous line (or type "x" in editable version)

x

© 2023 Liu M et al. *JAMA Network Open*

Very low credibility	Low credibility	Moderate credibility	High credibility
Very likely no effect modification Use overall effect for each subgroup	Likely no effect modification Use overall effect for each subgroup but note remaining uncertainty	Likely effect modification Use separate effects for each subgroup but note remaining uncertainty	Very likely effect modification Use separate effects for each subgroup

Comment:

© 2023 Liu M et al. *JAMA Network Open*

eTable 3.10 Credibility assessment of route of administration for Sever adverse event

1: Is the analysis of effect modification based on comparison within rather than between trials?

[ ] Completely between	[ ] Mostly between or unclear	[ ] Mostly within	[X] Completely within
Subgroup analysis or meta-regression comparing overall effects of each individual trial. This is typical for aggregate data meta-analysis.	*Subgroup analysis or meta-regression with most information coming from overall effects, but some trials providing within-trial subgroup information*	*Most trials providing within-trial subgroup information; or individual participant data analysis that combines within and between trial information*	*All trials providing within-trial subgroup information or individual participant data; and the analysis separates within from between trial information, e.g., meta-analysis of interactions*

Comment: Five trials provided within subgroups used for analysis.

2: For within-trial comparisons, is the effect modification similar from trial to trial? [ ] Not applicable: no or one within-RCT comparison

[ ] Definitely not similar	[ ] Probably not similar or unclear	[ ] Mostly similar	[X] Definitely similar
Effect modification reported for two or more trials and clearly different directions	*Effect modification not reported for individual trials or too imprecise to tell*	*Effect modification reported for two or more trials, mostly similar in direction, but considerable differences in magnitude*	*Effect modification reported for two or more trials, similar in direction, only some differences in magnitude*

Comment: The OR (i.e., the within-trial measure of effect modification) is always in the same direction.

3: For between-trial comparisons, is the number of trials large? [ ] Not applicable: no between RCT comparison

[ ] Very small	[X] Rather small or unclear	[ ] Rather large	[ ] Large
1 or 2 or in smallest subgroup; 5 or less in continuous meta-regression	*3-4 in smallest subgroup; 6-10 in continuous meta-regression*	*5-9 in smallest subgroup; 11 to 15 in continuous meta-regression*	*10 or more in smallest subgroup; more than 15 in continuous meta-regression*

Comment: Two RTCs is a rather larger number and two RCTs is a rather small number.

4: Was the direction of effect modification correctly hypothesised a priori?

[**X**] Definitely no	[X] Probably no or unclear	[ ] Probably yes	[ ] Definitely yes
Clearly post-hoc or results inconsistent with hypothesised direction biologically very implausible	*Vague hypothesis or hypothesised or direction unclear*	*No prior protocol available but unequivocal statement of a priori hypothesis with correct direction of effect modification*	*Prior protocol available and includes correct specification of direction of effect modification, e.g., based on a biologic rationale*

Comment: No information.

5: Does a test for interaction suggest that chance is an unlikely explanation of the apparent effect modification? (consider irrespective of number of effect modifiers)

[ ] Chance a very likely explanation	[ ] Chance a likely explanation or unclear	[ ] Chance may not explain	[X] Chance an unlikely explanation
Interaction or meta-regression p-value >0.05	*Interaction or meta-regression p-value ≤0.05 and >0.01, or no test of interaction reported and not*	*Interaction or meta-regression p-value ≤0.01 and >0.005*	*Interaction or meta-regression p-value ≤0.005*

© 2023 Liu M et al. *JAMA Network Open*

	computable		

Comment: The interaction P-value < 0.01.

6: Did the authors test only a small number of effect modifiers or consider the number in their statistical analysis?

[ ] Definitely no	[X] Probably no or unclear	[ ] Probably yes	[ ] Definitely yes
Explicitly exploratory analysis or large number of effect modifiers tested (e.g., greater than 10) and multiplicity not considered in analysis	*No mention of number or 4-10 effect modifiers tested and number not considered in analysis*	*No protocol available but unequivocal statement of 3 or fewer effect modifiers tested*	*Protocol available and 3 or fewer effect modifiers tested or number considered in analysis*

Comment: Two effect modifiers were tested in this review.

7: Did the authors use a random effects model? [ ] Not applicable

[ ] Definitely no	[ ] Probably no or unclear	[ ] Probably yes	[X] Definitely yes
Fixed (or common) effect or fixed effects model explicitly stated	*Probably fixed effect(s) model*	*Probably random (or mixed) effects*	*Random (or mixed) effects explicitly stated*

Comment: Yes, random effects model for combining the interaction estimates.

8: If the effect modifier is a continuous variable, were arbitrary cut points avoided? [X]not applicable: not continuous

[ ] Definitely no	[ ] Probably no or unclear	[ ] Probably yes	[ ] Definitely yes
Analysis based on exploratory cut point(s), e.g., picking cut point associated with highest interaction p-value	*Analysis based on cut point(s) of unclear origin*	*Analysis based on pre-specified cut point(s), e.g., suggested by prior RCT*	*Analysis based on the full continuum, e.g., assuming a linear or logarithmic relationship*

Comment:

9 Optional: Are there any additional considerations that may increase or decrease credibility? (manual section 3.9) [X] not applicable

	[ ] Yes, probably decrease<br>Biologically implausible<br>Expect similar severe critical<br>Opposite effects unlikely	[ ] Yes, probably increase

Comment:

10: How would you rate the overall credibility of the proposed effect modification?

The overall rating should be driven by the items that decrease credibility. The following provides a sensible strategy:

- All responses definitely or probably decrease credibility or unclear → very low
- Two or more responses definitely decrease credibility → maximum usually low even if all other responses satisfy credibility criteria
- One response definitely decreases credibility → maximum usually moderate even if all other responses satisfy credibility criteria
- Two responses probably decrease credibility → maximum usually moderate even if all other responses satisfy credibility criteria
- No response options definitely or probably decrease credibility → high very likely

Place a mark on the continuous line (or type "x" in editable version)

x

© 2023 Liu M et al. *JAMA Network Open*

Very low credibility	Low credibility	Moderate credibility	High credibility
Very likely no effect modification<br>Use overall effect for each subgroup	Likely no effect modification<br>Use overall effect for each subgroup but note remaining uncertainty	Likely effect modification<br>Use separate effects for each subgroup but note remaining uncertainty	Very likely effect modification<br>Use separate effects for each subgroup

Comment:

© 2023 Liu M et al. *JAMA Network Open*

eTable 3.11 Credibility assessment of different drugs for Sever adverse event

1: Is the analysis of effect modification based on comparison within rather than between trials?

[ ] Completely between	[ ] Mostly between or unclear	[ ] Mostly within	[X] Completely within
Subgroup analysis or meta-regression comparing overall effects of each individual trial. This is typical for aggregate data meta-analysis.	*Subgroup analysis or meta-regression with most information coming from overall effects, but some trials providing within-trial subgroup information*	*Most trials providing within-trial subgroup information; or individual participant data analysis that combines within and between trial information*	*All trials providing within-trial subgroup information or individual participant data; and the analysis separates within from between trial information, e.g., meta-analysis of interactions*

Comment: Five trials provided within subgroups used for analysis.

2: For within-trial comparisons, is the effect modification similar from trial to trial? [ ] Not applicable: no or one within-RCT comparison

[ ] Definitely not similar	[ ] Probably not similar or unclear	[ ] Mostly similar	[X] Definitely similar
Effect modification reported for two or more trials and clearly different directions	*Effect modification not reported for individual trials or too imprecise to tell*	*Effect modification reported for two or more trials, mostly similar in direction, but considerable differences in magnitude*	*Effect modification reported for two or more trials, similar in direction, only some differences in magnitude*

Comment: The ratio of OR (i.e., the within-trial measure of effect modification) is always in the same direction.

3: For between-trial comparisons, is the number of trials large? [ ] Not applicable: no between RCT comparison

[ ] Very small	[X] Rather small or unclear	[ ] Rather large	[ ] Large
1 or 2 or in smallest subgroup; 5 or less in continuous meta-regression	*3-4 in smallest subgroup; 6-10 in continuous meta-regression*	*5-9 in smallest subgroup; 11 to 15 in continuous meta-regression*	*10 or more in smallest subgroup; more than 15 in continuous meta-regression*

Comment: Three RTCs is a rather larger number and two RCTs is a rather small number.

4: Was the direction of effect modification correctly hypothesised a priori?

[ ] Definitely no	[X] Probably no or unclear	[ ] Probably yes	[ ] Definitely yes
Clearly post-hoc or results inconsistent with hypothesised direction biologically very implausible	*Vague hypothesis or hypothesised or direction unclear*	*No prior protocol available but unequivocal statement of a priori hypothesis with correct direction of effect modification*	*Prior protocol available and includes correct specification of direction of effect modification, e.g., based on a biologic rationale*

Comment: No information.

5: Does a test for interaction suggest that chance is an unlikely explanation of the apparent effect modification? (consider irrespective of number of effect modifiers)

[ ] Chance a very likely explanation	[ ] Chance a likely explanation or unclear	[ ] Chance may not explain	[X]Chance an unlikely explanation
Interaction or meta-regression p-value >0.05	*Interaction or meta-regression p-value ≤0.05 and >0.01, or no test of interaction reported and not computable*	*Interaction or meta-regression p-value ≤0.01 and >0.005*	*Interaction or meta-regression p-value ≤0.005*

Comment: The interaction P-value < 0.01.

© 2023 Liu M et al. *JAMA Network Open*

6: Did the authors test only a small number of effect modifiers or consider the number in their statistical analysis?

[ ] Definitely no	[X] Probably no or unclear	[ ] Probably yes	[ ] Definitely yes
Explicitly exploratory analysis or large number of effect modifiers tested (e.g., greater than 10) and multiplicity not considered in analysis	*No mention of number or 4-10 effect modifiers tested and number not considered in analysis*	*No protocol available but unequivocal statement of 3 or fewer effect modifiers tested*	*Protocol available and 3 or fewer effect modifiers tested or number considered in analysis*

Comment: Two effect modifiers were tested in this review.

7: Did the authors use a random effects model? [ ] Not applicable

[ ] Definitely no	[ ] Probably no or unclear	[ ] Probably yes	[X] Definitely yes
Fixed (or common) effect or fixed effects model explicitly stated	*Probably fixed effect(s) model*	*Probably random (or mixed) effects*	*Random (or mixed) effects explicitly stated*

Comment: Yes, random effects model for combining the interaction estimates.

8: If the effect modifier is a continuous variable, were arbitrary cut points avoided? [X] not applicable: not continuous

[ ] Definitely no	[ ] Probably no or unclear	[ ] Probably yes	[ ] Definitely yes
Analysis based on exploratory cut point(s), e.g., picking cut point associated with highest interaction p-value	*Analysis based on cut point(s) of unclear origin*	*Analysis based on pre-specified cut point(s), e.g., suggested by prior RCT*	*Analysis based on the full continuum, e.g., assuming a linear or logarithmic relationship*

Comment:

9 Optional: Are there any additional considerations that may increase or decrease credibility? (manual section 3.9) [X] not applicable

	[ ] Yes, probably decrease	[ ] Yes, probably increase
	Biologically implausible<br>Expect similar severe critical<br>Opposite effects unlikely	

Comment:

10: How would you rate the overall credibility of the proposed effect modification?

The overall rating should be driven by the items that decrease credibility. The following provides a sensible strategy:

- All responses definitely or probably decrease credibility or unclear → very low
- Two or more responses definitely decrease credibility → maximum usually low even if all other responses satisfy credibility criteria
- One response definitely decreases credibility → maximum usually moderate even if all other responses satisfy credibility criteria
- Two responses probably decrease credibility → maximum usually moderate even if all other responses satisfy credibility criteria
- No response options definitely or probably decrease credibility → high very likely

Place a mark on the continuous line (or type "x" in editable version)

X

Very low credibility	Low credibility	Moderate credibility	High credibility

© 2023 Liu M et al. *JAMA Network Open*

Very likely no effect modification	Likely no effect modification	Likely effect modification	Very likely effect modification
Use overall effect for each subgroup	Use overall effect for each subgroup but note remaining uncertainty	Use separate effects for each subgroup but note remaining uncertainty	Use separate effects for each subgroup

Comment:

© 2023 Liu M et al. *JAMA Network Open*

eTable 3.12 Credibility assessment of route of administration for discontinuation due to adverse event

1: Is the analysis of effect modification based on comparison within rather than between trials?

[ ] Completely between	[ ] Mostly between or unclear	[ ] Mostly within	[X] Completely within
Subgroup analysis or meta-regression comparing overall effects of each individual trial. This is typical for aggregate data meta-analysis.	*Subgroup analysis or meta-regression with most information coming from overall effects, but some trials providing within-trial subgroup information*	*Most trials providing within-trial subgroup information; or individual participant data analysis that combines within and between trial information*	*All trials providing within-trial subgroup information or individual participant data; and the analysis separates within from between trial information, e.g., meta-analysis of interactions*

Comment: Five trials provided within subgroups used for analysis.

2: For within-trial comparisons, is the effect modification similar from trial to trial? [ ] Not applicable: no or one within-RCT comparison

[ ] Definitely not similar	[ ] Probably not similar or unclear	[ ] Mostly similar	[X] Definitely similar
Effect modification reported for two or more trials and clearly different directions	*Effect modification not reported for individual trials or too imprecise to tell*	*Effect modification reported for two or more trials, mostly similar in direction, but considerable differences in magnitude*	*Effect modification reported for two or more trials, similar in direction, only some differences in magnitude*

Comment: The OR (i.e., the within-trial measure of effect modification) is always in the same direction.

3: For between-trial comparisons, is the number of trials large? [ ] Not applicable: no between RCT comparison

[ ] Very small	[X] Rather small or unclear	[ ] Rather large	[ ] Large
1 or 2 or in smallest subgroup; 5 or less in continuous meta-regression	*3-4 in smallest subgroup; 6-10 in continuous meta-regression*	*5-9 in smallest subgroup; 11 to 15 in continuous meta-regression*	*10 or more in smallest subgroup; more than 15 in continuous meta-regression*

Comment: Two RTCs is a rather larger number and two RCTs is a rather small number.

4: Was the direction of effect modification correctly hypothesised a priori?

[**X**] Definitely no	[X] Probably no or unclear	[ ] Probably yes	[ ] Definitely yes
Clearly post-hoc or results inconsistent with hypothesised direction biologically very implausible	*Vague hypothesis or hypothesised or direction unclear*	*No prior protocol available but unequivocal statement of a priori hypothesis with correct direction of effect modification*	*Prior protocol available and includes correct specification of direction of effect modification, e.g., based on a biologic rationale*

Comment: No information.

5: Does a test for interaction suggest that chance is an unlikely explanation of the apparent effect modification? (consider irrespective of number of effect modifiers)

[ ] Chance a very likely explanation	[ ] Chance a likely explanation or unclear	[ ] Chance may not explain	[X] Chance an unlikely explanation
Interaction or meta-regression p-value >0.05	*Interaction or meta-regression p-value ≤0.05 and >0.01, or no test of interaction reported and not computable*	*Interaction or meta-regression p-value ≤0.01 and >0.005*	*Interaction or meta-regression p-value ≤0.005*

Comment: The interaction P-value < 0.01.

© 2023 Liu M et al. *JAMA Network Open*

6: Did the authors test only a small number of effect modifiers or consider the number in their statistical analysis?

[ ] Definitely no	[X] Probably no or unclear	[ ] Probably yes	[ ] Definitely yes
Explicitly exploratory analysis or large number of effect modifiers tested (e.g., greater than 10) and multiplicity not considered in analysis	*No mention of number or 4-10 effect modifiers tested and number not considered in analysis*	*No protocol available but unequivocal statement of 3 or fewer effect modifiers tested*	*Protocol available and 3 or fewer effect modifiers tested or number considered in analysis*

Comment: Two effect modifiers were tested in this review.

7: Did the authors use a random effects model? [ ] Not applicable

[ ] Definitely no	[ ] Probably no or unclear	[ ] Probably yes	[X] Definitely yes
Fixed (or common) effect or fixed effects model explicitly stated	*Probably fixed effect(s) model*	*Probably random (or mixed) effects*	*Random (or mixed) effects explicitly stated*

Comment: Yes, random effects model for combining the interaction estimates.

8: If the effect modifier is a continuous variable, were arbitrary cut points avoided? [X]not applicable: not continuous

[ ] Definitely no	[ ] Probably no or unclear	[ ] Probably yes	[ ] Definitely yes
Analysis based on exploratory cut point(s), e.g., picking cut point associated with highest interaction p-value	*Analysis based on cut point(s) of unclear origin*	*Analysis based on pre-specified cut point(s), e.g., suggested by prior RCT*	*Analysis based on the full continuum, e.g., assuming a linear or logarithmic relationship*

Comment:

9 Optional: Are there any additional considerations that may increase or decrease credibility? (manual section 3.9) [X] not applicable

[ ] Yes, probably decrease	[ ] Yes, probably increase
Biologically implausible<br>Expect similar severe critical<br>Opposite effects unlikely	

Comment:

10: How would you rate the overall credibility of the proposed effect modification?

The overall rating should be driven by the items that decrease credibility. The following provides a sensible strategy:

- All responses definitely or probably decrease credibility or unclear → very low
- Two or more responses definitely decrease credibility → maximum usually low even if all other responses satisfy credibility criteria
- One response definitely decreases credibility → maximum usually moderate even if all other responses satisfy credibility criteria
- Two responses probably decrease credibility → maximum usually moderate even if all other responses satisfy credibility criteria
- No response options definitely or probably decrease credibility → high very likely

Place a mark on the continuous line (or type "x" in editable version)

X

**Very low credibility**	**Low credibility**	**Moderate credibility**	**High credibility**

© 2023 Liu M et al. *JAMA Network Open*

Very likely no effect modification<br>Use overall effect for each subgroup	Likely no effect modification<br>Use overall effect for each subgroup but note remaining uncertainty	Likely effect modification<br>Use separate effects for each subgroup but note remaining uncertainty	Very likely effect modification<br>Use separate effects for each subgroup

Comment:

© 2023 Liu M et al. *JAMA Network Open*

eTable 3.13 Credibility assessment of different drugs for discontinuation due to adverse event

1: Is the analysis of effect modification based on comparison within rather than between trials?

[ ] Completely between	[ ] Mostly between or unclear	[ ] Mostly within	[X] Completely within
Subgroup analysis or meta-regression comparing overall effects of each individual trial. This is typical for aggregate data meta-analysis.	*Subgroup analysis or meta-regression with most information coming from overall effects, but some trials providing within-trial subgroup information*	*Most trials providing within-trial subgroup information; or individual participant data analysis that combines within and between trial information*	*All trials providing within-trial subgroup information or individual participant data; and the analysis separates within from between trial information, e.g., meta-analysis of interactions*

Comment: Five trials provided within subgroups used for analysis.

2: For within-trial comparisons, is the effect modification similar from trial to trial? [ ] Not applicable: no or one within-RCT comparison

[ ] Definitely not similar	[ ] Probably not similar or unclear	[ ] Mostly similar	[X] Definitely similar
Effect modification reported for two or more trials and clearly different directions	*Effect modification not reported for individual trials or too imprecise to tell*	*Effect modification reported for two or more trials, mostly similar in direction, but considerable differences in magnitude*	*Effect modification reported for two or more trials, similar in direction, only some differences in magnitude*

Comment: The ratio of OR (i.e., the within-trial measure of effect modification) is always in the same direction.

3: For between-trial comparisons, is the number of trials large? [ ] Not applicable: no between RCT comparison

[ ] Very small	[X] Rather small or unclear	[ ] Rather large	[ ] Large
1 or 2 or in smallest subgroup; 5 or less in continuous meta-regression	*3-4 in smallest subgroup; 6-10 in continuous meta-regression*	*5-9 in smallest subgroup; 11 to 15 in continuous meta-regression*	*10 or more in smallest subgroup; more than 15 in continuous meta-regression*

Comment: Three RTCs is a rather larger number and two RCTs is a rather small number.

4: Was the direction of effect modification correctly hypothesised a priori?

[ ] Definitely no	[X] Probably no or unclear	[ ] Probably yes	[ ] Definitely yes
Clearly post-hoc or results inconsistent with hypothesised direction or biologically very implausible	*Vague hypothesis or hypothesised direction unclear*	*No prior protocol available but unequivocal statement of a priori hypothesis with correct direction of effect modification*	*Prior protocol available and includes correct specification of direction of effect modification, e.g., based on a biologic rationale*

Comment: No information.

5: Does a test for interaction suggest that chance is an unlikely explanation of the apparent effect modification? (consider irrespective of number of effect modifiers)

[ ] Chance a very likely explanation	[ ] Chance a likely explanation or unclear	[ ] Chance may not explain	[X]Chance an unlikely explanation
Interaction or meta-regression p-value >0.05	*Interaction or meta-regression p-value ≤0.05 and >0.01, or no test of interaction reported and not*	*Interaction or meta-regression p-value ≤0.01 and >0.005*	*Interaction or meta-regression p-value ≤0.005*

© 2023 Liu M et al. *JAMA Network Open*

	computable		

Comment: The interaction P-value < 0.01.

6: Did the authors test only a small number of effect modifiers or consider the number in their statistical analysis?

[ ] Definitely no	[X] Probably no or unclear	[ ] Probably yes	[ ] Definitely yes
Explicitly exploratory analysis or large number of effect modifiers tested (e.g., greater than 10) and multiplicity not considered in analysis	*No mention of number or 4-10 effect modifiers tested and number not considered in analysis*	*No protocol available but unequivocal statement of 3 or fewer effect modifiers tested*	*Protocol available and 3 or fewer effect modifiers tested or number considered in analysis*

Comment: Two effect modifiers were tested in this review.

7: Did the authors use a random effects model? [ ] Not applicable

[ ] Definitely no	[ ] Probably no or unclear	[ ] Probably yes	[X] Definitely yes
Fixed (or common) effect or fixed effects model explicitly stated	*Probably fixed effect(s) model*	*Probably random (or mixed) effects*	*Random (or mixed) effects explicitly stated*

Comment: Yes, random effects model for combining the interaction estimates.

8: If the effect modifier is a continuous variable, were arbitrary cut points avoided? [X] not applicable: not continuous

[ ] Definitely no	[ ] Probably no or unclear	[ ] Probably yes	[ ] Definitely yes
Analysis based on exploratory cut point(s), e.g., picking cut point associated with highest interaction p-value	*Analysis based on cut point(s) of unclear origin*	*Analysis based on pre-specified cut point(s), e.g., suggested by prior RCT*	*Analysis based on the full continuum, e.g., assuming a linear or logarithmic relationship*

Comment:

9 Optional: Are there any additional considerations that may increase or decrease credibility? (manual section 3.9) [X] not applicable

	[ ] Yes, probably decrease<br>Biologically implausible<br>Expect similar severe critical<br>Opposite effects unlikely	[ ] Yes, probably increase

Comment:

10: How would you rate the overall credibility of the proposed effect modification?

The overall rating should be driven by the items that decrease credibility. The following provides a sensible strategy:

- All responses definitely or probably decrease credibility or unclear → very low
- Two or more responses definitely decrease credibility → maximum usually low even if all other responses satisfy credibility criteria
- One response definitely decreases credibility → maximum usually moderate even if all other responses satisfy credibility criteria
- Two responses probably decrease credibility → maximum usually moderate even if all other responses satisfy credibility criteria
- No response options definitely or probably decrease credibility → high very likely

Place a mark on the continuous line (or type "x" in editable version)

x

© 2023 Liu M et al. *JAMA Network Open*

Very low credibility	Low credibility	Moderate credibility	High credibility
Very likely no effect modification<br>Use overall effect for each subgroup	Likely no effect modification<br>Use overall effect for each subgroup but note remaining uncertainty	Likely effect modification<br>Use separate effects for each subgroup but note remaining uncertainty	Very likely effect modification<br>Use separate effects for each subgroup

Comment:

© 2023 Liu M et al. *JAMA Network Open*

eFigure 1 Subgroup analysis by route of administration of JAK inhibitors versus placebo on change from baseline of SALT scores

Study	JAK inhibitors Total	JAK inhibitors Mean	JAK inhibitors SD	Placebo Total	Placebo Mean	Placebo SD	MD	95%CI	Weight
Oral									
King(2021a)	95	−41.00	20.21	47	−1.40	11.75	−39.60	[−44.87; −34.33]	17.7%
King(2021b)	54	−53.20	9.24	28	−11.70	7.80	−41.50	[−45.30; −37.70]	22.2%
King(2022b BRAVE−AA1)	465	−41.40	7.61	189	−9.00	3.10	−32.40	[−33.22; −31.58]	30.2%
King(2022b BRAVE−AA2)	390	−41.20	9.60	156	−4.30	2.80	−36.90	[−37.95; −35.85]	29.9%
**Hartung−Knapp−Sidik−Jonkman**	**1004**			**420**			**−36.80**	**[−39.57; −34.02]**	**100.0%**
Heterogeneity: $I^2 = 95\%$, $\tau^2 = 6.0215$, $p < 0.01$									
External									
Mikhaylov(2022)	20	−3.80	9.30	11	−3.40	17.10	−0.40	[−11.30; 10.50]	100.0%

Heterogeneity: $I^2 = 96\%$, $\tau^2 = 9.7674$, $p < 0.01$

Test for subgroup differences: $\chi^2_1 = 40.24$, df = 1 ($p < 0.01$)

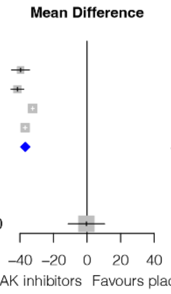

© 2023 Liu M et al. *JAMA Network Open*

eFigure 2 Subgroup analysis by different drugs of JAK inhibitors versus placebo on change from baseline of SALT scores

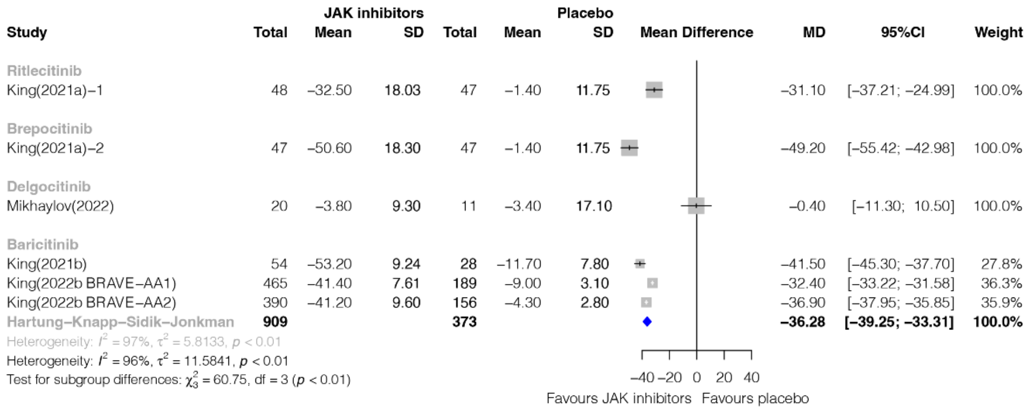

© 2023 Liu M et al. *JAMA Network Open*

eFigure 3 Subgroup analysis by route of administration of JAK inhibitors versus placebo on SALT 50

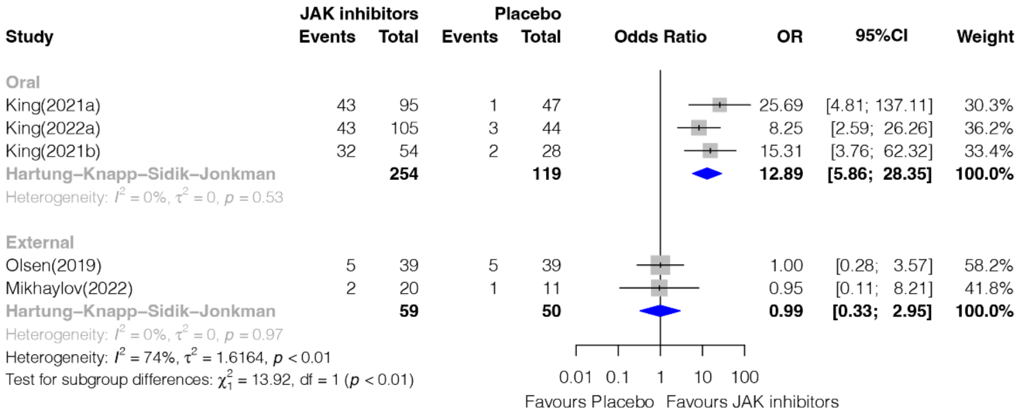

© 2023 Liu M et al. *JAMA Network Open*

eFigure 4 Subgroup analysis by different drugs of JAK inhibitors versus placebo on SALT 50

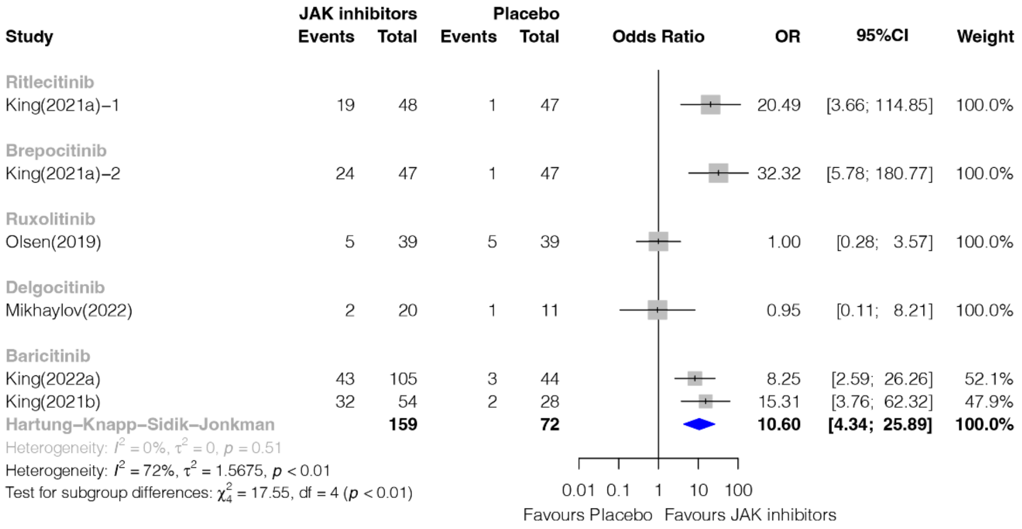

© 2023 Liu M et al. *JAMA Network Open*

eFigure 5 Subgroup analysis by route of administration of JAK inhibitors versus placebo on SALT 90

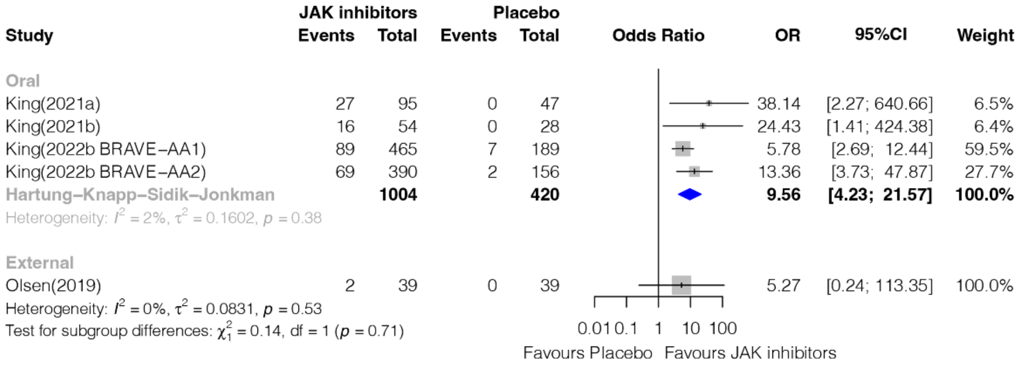

© 2023 Liu M et al. *JAMA Network Open*

eFigure 6 Subgroup analysis by different drugs of JAK inhibitors versus placebo on SALT 90

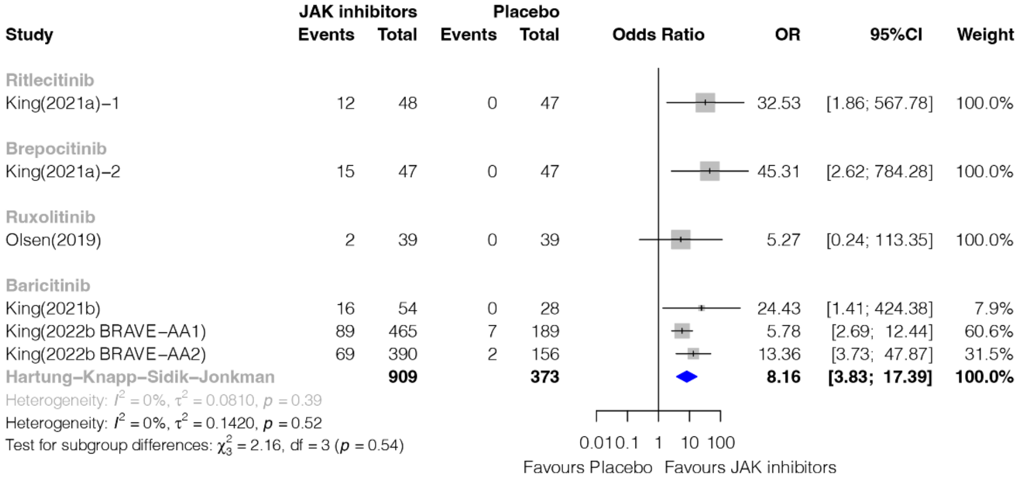

© 2023 Liu M et al. *JAMA Network Open*

eFigure 7 Subgroup analysis by different drugs of JAK inhibitors versus placebo on SALT score ≤ 20

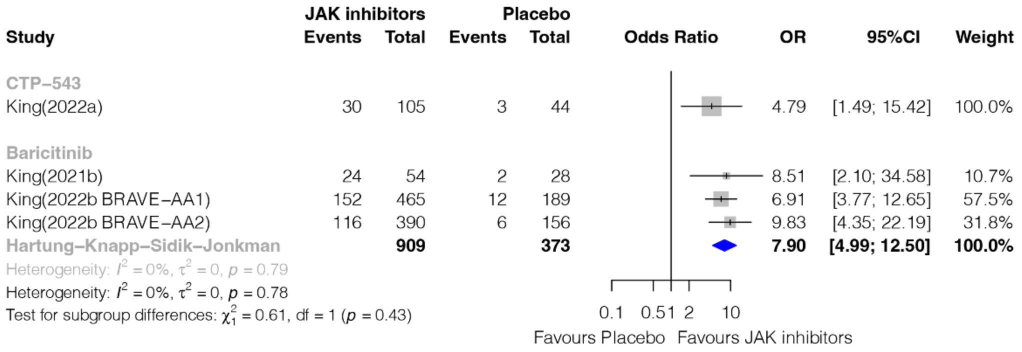

© 2023 Liu M et al. *JAMA Network Open*

eFigure 8 Subgroup analysis by route of administration of JAK inhibitors versus placebo on Treatment-related adverse event

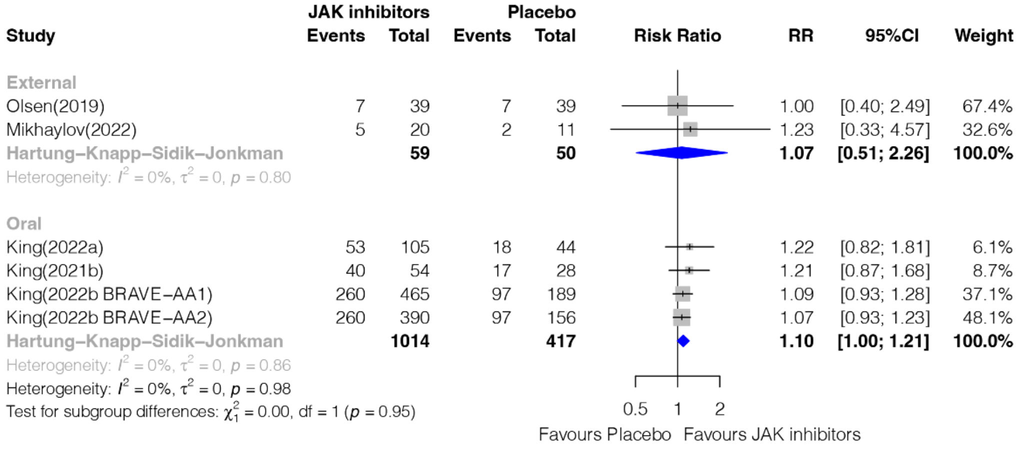

© 2023 Liu M et al. *JAMA Network Open*

eFigure 9 Subgroup analysis by different drugs of JAK inhibitors versus placebo on Treatment-related adverse event

Study	JAK inhibitors Events	JAK inhibitors Total	Placebo Events	Placebo Total	RR	95%CI	Weight
**Ruxolitinib**							
Olsen(2019)	7	39	7	39	1.00	[0.40; 2.49]	100.0%
**Delgocitinib**							
Mikhaylov(2022)	5	20	2	11	1.23	[0.33; 4.57]	100.0%
**CTP−543**							
King(2022a)	53	105	18	44	1.22	[0.82; 1.81]	100.0%
**Baricitinib**							
King(2021b)	40	54	17	28	1.21	[0.87; 1.68]	9.3%
King(2022b BRAVE−AA1)	260	465	97	189	1.09	[0.93; 1.28]	39.5%
King(2022b BRAVE−AA2)	260	390	97	156	1.07	[0.93; 1.23]	51.2%
**Hartung−Knapp−Sidik−Jonkman**		**909**		**373**	**1.09**	**[0.99; 1.21]**	**100.0%**

Heterogeneity: $I^2 = 0\%$, $\tau^2 = 0$, $p = 0.80$

Heterogeneity: $I^2 = 0\%$, $\tau^2 = 0$, $p = 0.98$

Test for subgroup differences: $\chi^2_3 = 0.36$, df = 3 ($p = 0.95$)

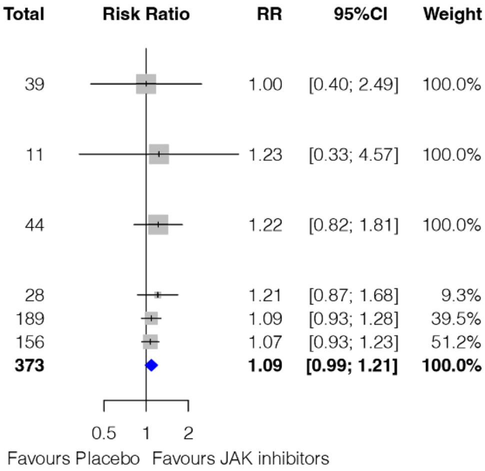

© 2023 Liu M et al. *JAMA Network Open*

eFigure 10 Subgroup analysis by route of administration of JAK inhibitors versus placebo on severe adverse event

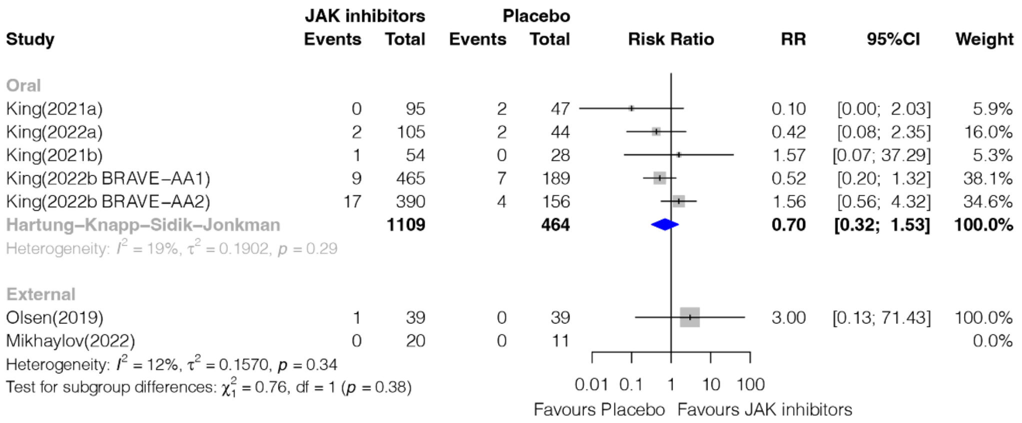

© 2023 Liu M et al. *JAMA Network Open*

eFigure 11 Subgroup analysis by different drugs of JAK inhibitors versus placebo on severe adverse event

Study	JAK inhibitors Events	JAK inhibitors Total	Placebo Events	Placebo Total	RR	95%CI	Weight
Ritlecitinib							
King(2021a)−1	0	48	2	47	0.20	[0.01; 3.97]	100.0%
Brepocitinib							
King(2021a)−2	0	47	2	47	0.20	[0.01; 4.06]	100.0%
Ruxolitinib							
Olsen(2019)	1	39	0	39	3.00	[0.13; 71.43]	100.0%
Delgocitinib							
Mikhaylov(2022)	0	20	0	11			−−
CTP−543							
King(2022a)	2	105	2	44	0.42	[0.08; 2.35]	100.0%
Baricitinib							
King(2021b)	1	54	0	28	1.57	[0.07; 37.29]	6.3%
King(2022b BRAVE−AA1)	9	465	7	189	0.52	[0.20; 1.32]	49.3%
King(2022b BRAVE−AA2)	17	390	4	156	1.56	[0.56; 4.32]	44.4%
**Hartung−Knapp−Sidik−Jonkman**		**909**		**373**	**0.92**	**[0.36; 2.36]**	**100.0%**

Heterogeneity: $I^2 = 22\%$, $\tau^2 = 0.2552$, $p = 0.28$

Heterogeneity: $I^2 = 0\%$, $\tau^2 = 0.1191$, $p = 0.49$

Test for subgroup differences: $\chi^2_4 = 2.82$, df = 4 ($p = 0.59$)

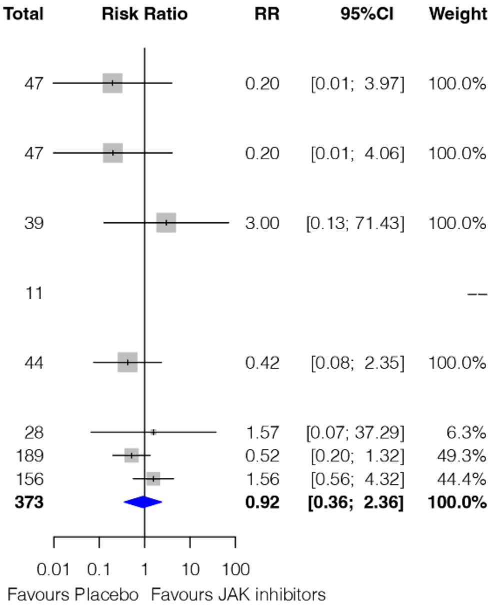

© 2023 Liu M et al. *JAMA Network Open*

eFigure 12 Subgroup analysis by route of administration of JAK inhibitors versus placebo on discontinuation due to adverse event

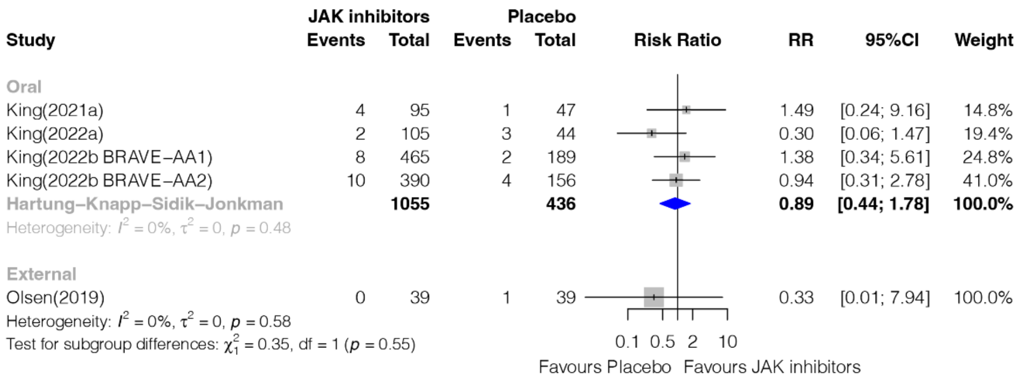

© 2023 Liu M et al. *JAMA Network Open*

eFigure 13 Subgroup analysis by different drugs of JAK inhibitors versus placebo on discontinuation due to adverse event

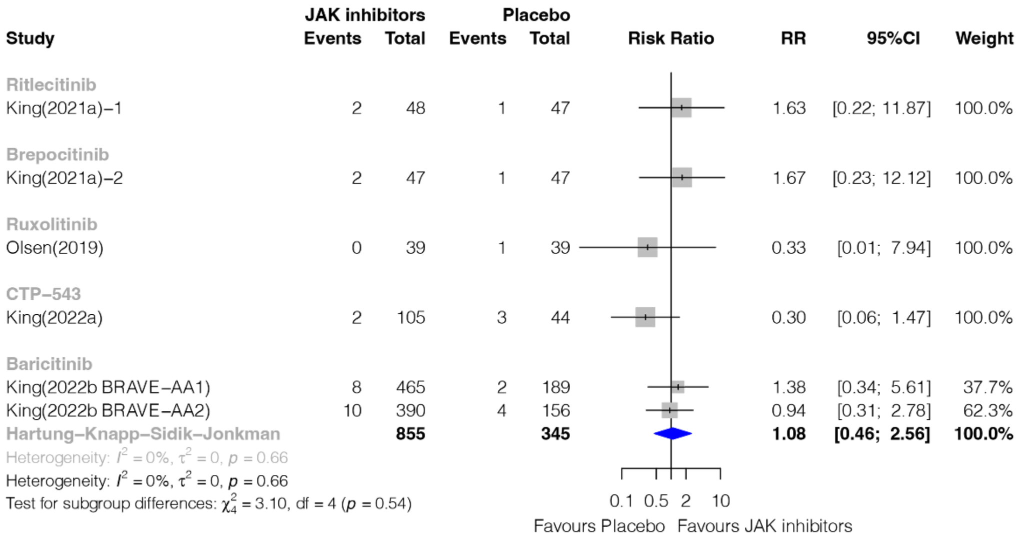

© 2023 Liu M et al. *JAMA Network Open*

eFigure 14 Sensitivity analysis for change from baseline of SALT scores used the DerSimonian-Laird random effect model

	JAK inhibitors			Placebo					
Study	Total	Mean	SD	Total	Mean	SD	MD	95%CI	Weight
King(2021a)	95	−41.00	20.21	47	−1.40	11.75	−39.60	[−44.87; −34.33]	18.5%
Mikhaylov(2022)	20	−3.80	9.30	11	−3.40	17.10	−0.40	[−11.30; 10.50]	9.7%
King(2021b)	54	−53.20	9.24	28	−11.70	7.80	−41.50	[−45.30; −37.70]	21.3%
King(2022b BRAVE−AA1)	465	−41.40	7.61	189	−9.00	3.10	−32.40	[−33.22; −31.58]	25.3%
King(2022b BRAVE−AA2)	390	−41.20	9.60	156	−4.30	2.80	−36.90	[−37.95; −35.85]	25.1%
**DerSimonian−Laird**	**1024**			**431**			**−33.69**	**[−38.01; −29.36]**	**100.0%**

Heterogeneity: $I^2 = 96\%$, $\tau^2 = 19.0942$, $p < 0.01$

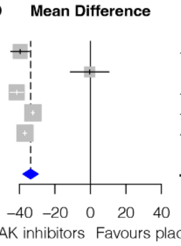

© 2023 Liu M et al. *JAMA Network Open*

eFigure 15 Sensitivity analysis for SALT scores (SALT 50, SALT 90, SALT score ≤ 10 and SALT score ≤ 20) used the DerSimonian-Laird random effect model

Study	JAK inhibitors Events	JAK inhibitors Total	Placebo Events	Placebo Total	OR	95%CI	Weight
**SALT 50**							
King(2021a)	43	95	1	47	25.69	[4.81; 137.11]	16.0%
Olsen(2019)	5	39	5	39	1.00	[0.28; 3.57]	24.5%
Mikhaylov(2022)	2	20	1	11	0.95	[0.11; 8.21]	10.3%
King(2022a)	43	105	3	44	8.25	[2.59; 26.26]	28.1%
King(2021b)	32	54	2	28	15.31	[3.76; 62.32]	21.2%
**DerSimonian...Laird**		**313**		**169**	**5.23**	**[1.44; 18.96]**	**100.0%**
Heterogeneity: $I^2 = 74\%$, $\tau^2 = 1.5504$, $p < 0.01$							
**SALT 90**							
King(2021a)	27	95	0	47	38.14	[2.27; 640.66]	7.2%
Olsen(2019)	2	39	0	39	5.27	[0.24; 113.35]	6.1%
King(2021b)	16	54	0	28	24.43	[1.41; 424.38]	7.0%
King(2022b BRAVE−AA1)	89	465	7	189	5.78	[2.69; 12.44]	52.1%
King(2022b BRAVE−AA2)	69	390	2	156	13.36	[3.73; 47.87]	27.6%
**DerSimonian...Laird**		**1043**		**459**	**8.15**	**[4.42; 15.03]**	**100.0%**
Heterogeneity: $I^2 = 0\%$, $\tau^2 = 0$, $p = 0.53$							
**SALT score ≤ 10**							
King(2021b)	18	54	0	28	28.89	[1.67; 500.16]	7.8%
King(2022b BRAVE−AA1)	103	465	8	189	6.10	[2.96; 12.55]	61.3%
King(2022b BRAVE−AA2)	79	390	2	156	15.77	[4.41; 56.37]	30.8%
**DerSimonian...Laird**		**909**		**373**	**8.90**	**[4.18; 18.95]**	**100.0%**
Heterogeneity: $I^2 = 17\%$, $\tau^2 = 0.0950$, $p = 0.30$							
**SALT score ≤ 20**							
King(2022a)	30	105	3	44	4.79	[1.49; 15.42]	18.6%
King(2021b)	24	54	2	28	8.51	[2.10; 34.58]	14.3%
King(2022b BRAVE−AA1)	152	465	12	189	6.91	[3.77; 12.65]	38.1%
King(2022b BRAVE−AA2)	116	390	6	156	9.83	[4.35; 22.19]	29.1%
**DerSimonian...Laird**		**1014**		**417**	**7.39**	**[4.82; 11.33]**	**100.0%**
Heterogeneity: $I^2 = 0\%$, $\tau^2 = 0$, $p = 0.78$							
Heterogeneity: $I^2 = 30\%$, $\tau^2 = 0.1540$, $p = 0.11$							

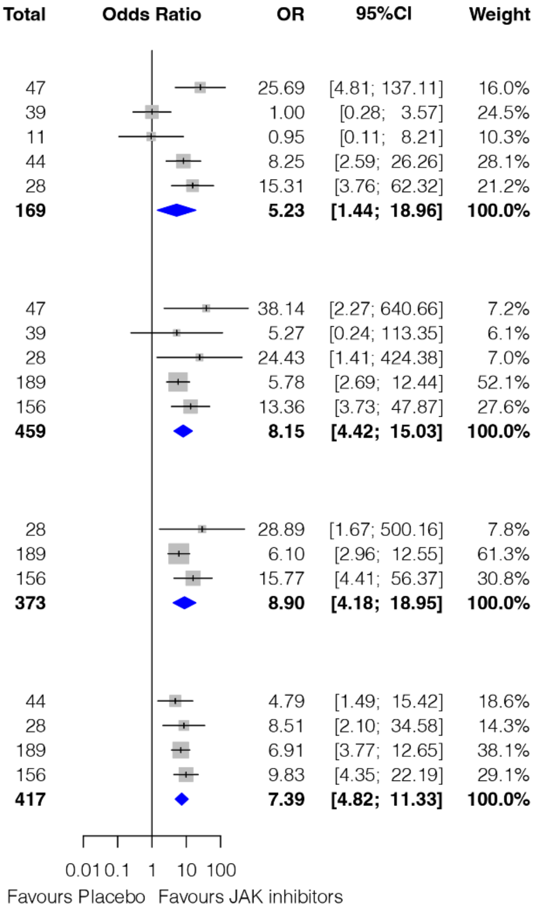

© 2023 Liu M et al. *JAMA Network Open*

eFigure 16 Sensitivity analysis for safety used the DerSimonian-Laird random effect model

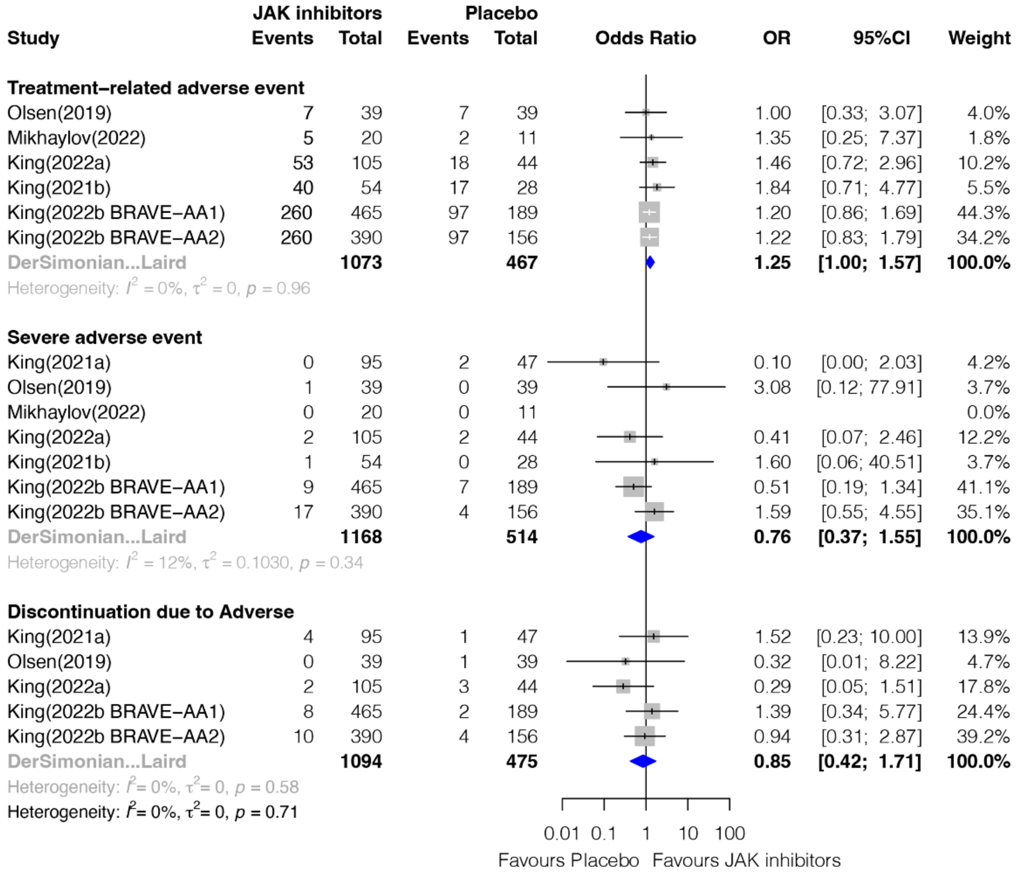

© 2023 Liu M et al. *JAMA Network Open*

eFigure 17 Sensitivity analysis for change from baseline of SALT scores after excluded high risk bias of studies

	JAK inhibitors			Placebo					
**Study**	**Total**	**Mean**	**SD**	**Total**	**Mean**	**SD**	**MD**	**95%CI**	**Weight**
King(2021b)	54	−53.20	9.24	28	−11.70	7.80	−41.50	[−45.30; −37.70]	24.0%
King(2022b BRAVE−AA1)	465	−41.40	7.61	189	−9.00	3.10	−32.40	[−33.22; −31.58]	38.3%
King(2022b BRAVE−AA2)	390	−41.20	9.60	156	−4.30	2.80	−36.90	[−37.95; −35.85]	37.6%
**Hartung−Knapp−Sidik−Jonkman**	**909**			**373**			**−36.28**	**[−39.25; −33.31]**	**100.0%**

Heterogeneity: $I^2 = 97\%$, $\tau^2 = 5.8133$, $p < 0.01$

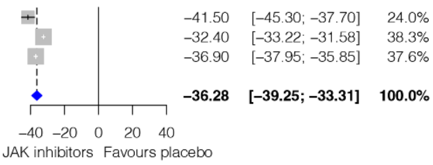

© 2023 Liu M et al. *JAMA Network Open*

eFigure 18 Sensitivity analysis for SALT scores (SALT 50, SALT 90, SALT score ≤ 10 and SALT score ≤ 20) after excluded high risk bias of studies

Study	JAK inhibitors Events	JAK inhibitors Total	Placebo Events	Placebo Total	OR	95%CI	Weight
**SALT 50**							
Olsen(2019)	5	39	5	39	1.00	[0.28; 3.57]	33.1%
King(2022a)	43	105	3	44	8.25	[2.59; 26.26]	39.2%
King(2021b)	32	54	2	28	15.31	[3.76; 62.32]	27.7%
**Hartung–Knapp–Sidik–Jonkman**	**198**		**111**		**4.95**	**[1.36; 17.98]**	**100.0%**
Heterogeneity: $I^2 = 78\%$, $\tau^2 = 0.8764$, $p < 0.01$							
**SALT 90**							
King(2021a)	27	95	0	47	38.14	[2.27; 640.66]	5.6%
Olsen(2019)	2	39	0	39	5.27	[0.24; 113.35]	4.8%
King(2021b)	16	54	0	28	24.43	[1.41; 424.38]	5.5%
King(2022b BRAVE–AA1)	89	465	7	189	5.78	[2.69; 12.44]	59.0%
King(2022b BRAVE–AA2)	69	390	2	156	13.36	[3.73; 47.87]	25.1%
**Hartung–Knapp–Sidik–Jonkman**	**1043**		**459**		**8.15**	**[4.42; 15.03]**	**100.0%**
Heterogeneity: $I^2 = 0\%$, $\tau^2 = 0$, $p = 0.53$							
**SALT score ≤ 10**							
King(2021b)	18	54	0	28	28.89	[1.67; 500.16]	5.8%
King(2022b BRAVE–AA1)	103	465	8	189	6.10	[2.96; 12.55]	67.7%
King(2022b BRAVE–AA2)	79	390	2	156	15.77	[4.41; 56.37]	26.5%
**Hartung–Knapp–Sidik–Jonkman**	**909**		**373**		**8.17**	**[4.42; 15.08]**	**100.0%**
Heterogeneity: $I^2 = 17\%$, $\tau^2 = 0$, $p = 0.30$							
**SALT score ≤ 20**							
King(2022a)	30	105	3	44	4.79	[1.49; 15.42]	15.7%
King(2021b)	24	54	2	28	8.51	[2.10; 34.58]	11.4%
King(2022b BRAVE–AA1)	152	465	12	189	6.91	[3.77; 12.65]	44.2%
King(2022b BRAVE–AA2)	116	390	6	156	9.83	[4.35; 22.19]	28.7%
**Hartung–Knapp–Sidik–Jonkman**	**1014**		**417**		**7.39**	**[4.82; 11.33]**	**100.0%**
Heterogeneity: $I^2 = 0\%$, $\tau^2 = 0$, $p = 0.78$							
Heterogeneity: $I^2 = 19\%$, $\tau^2 = 0.0488$, $p = 0.24$							

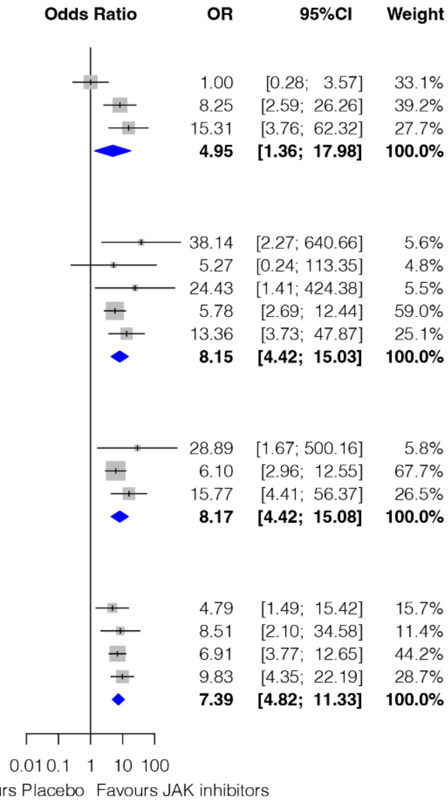

© 2023 Liu M et al. *JAMA Network Open*

eFigure 19 Sensitivity analysis for safety after excluded high risk bias of studies

Study	JAK inhibitors Events	JAK inhibitors Total	Placebo Events	Placebo Total	OR	95%CI	Weight
**Treatment–related adverse event**							
Olsen(2019)	7	39	7	39	1.00	[0.33; 3.07]	4.1%
King(2022a)	53	105	18	44	1.46	[0.72; 2.96]	10.4%
King(2021b)	40	54	17	28	1.84	[0.71; 4.77]	5.6%
King(2022b BRAVE–AA1)	260	465	97	189	1.20	[0.86; 1.69]	45.1%
King(2022b BRAVE–AA2)	260	390	97	156	1.22	[0.83; 1.79]	34.8%
**Hartung–Knapp–Sidik–Jonkman**		**1053**		**456**	**1.25**	**[1.00; 1.57]**	**100.0%**
Heterogeneity: $I^2 = 0\%$, $\tau^2 = 0$, $p = 0.91$							
**Severe adverse event**							
Olsen(2019)	1	39	0	39	3.08	[0.12; 77.91]	3.9%
King(2022a)	2	105	2	44	0.41	[0.07; 2.46]	12.7%
King(2021b)	1	54	0	28	1.60	[0.06; 40.51]	3.9%
King(2022b BRAVE–AA1)	9	465	7	189	0.51	[0.19; 1.34]	42.9%
King(2022b BRAVE–AA2)	17	390	4	156	1.59	[0.55; 4.55]	36.6%
**Hartung–Knapp–Sidik–Jonkman**		**1053**		**456**	**0.84**	**[0.44; 1.59]**	**100.0%**
Heterogeneity: $I^2 = 0\%$, $\tau^2 = 0$, $p = 0.43$							
**Discontinuation due to Adverse**							
Olsen(2019)	0	39	1	39	0.32	[0.01; 8.22]	5.5%
King(2022a)	2	105	3	44	0.29	[0.05; 1.51]	20.7%
King(2022b BRAVE–AA1)	8	465	2	189	1.39	[0.34; 5.77]	28.3%
King(2022b BRAVE–AA2)	10	390	4	156	0.94	[0.31; 2.87]	45.5%
**Hartung–Knapp–Sidik–Jonkman**		**999**		**428**	**0.77**	**[0.36; 1.65]**	**100.0%**
Heterogeneity: $I^2 = 0\%$, $\tau^2 = 0$, $p = 0.49$							
Heterogeneity: $I^2 = 0\%$, $\tau^2 = 0$, $p = 0.71$							

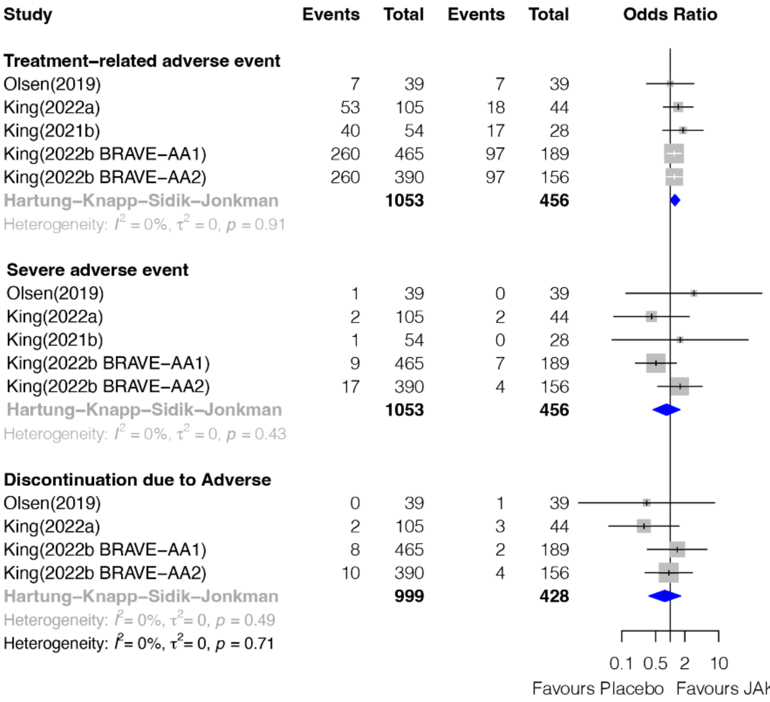

© 2023 Liu M et al. *JAMA Network Open*

eFigure 20 Sensitivity analysis for SALT scores (SALT 50, SALT 90, SALT score ≤ 10 and SALT score ≤ 20) after excluded zero-events studies

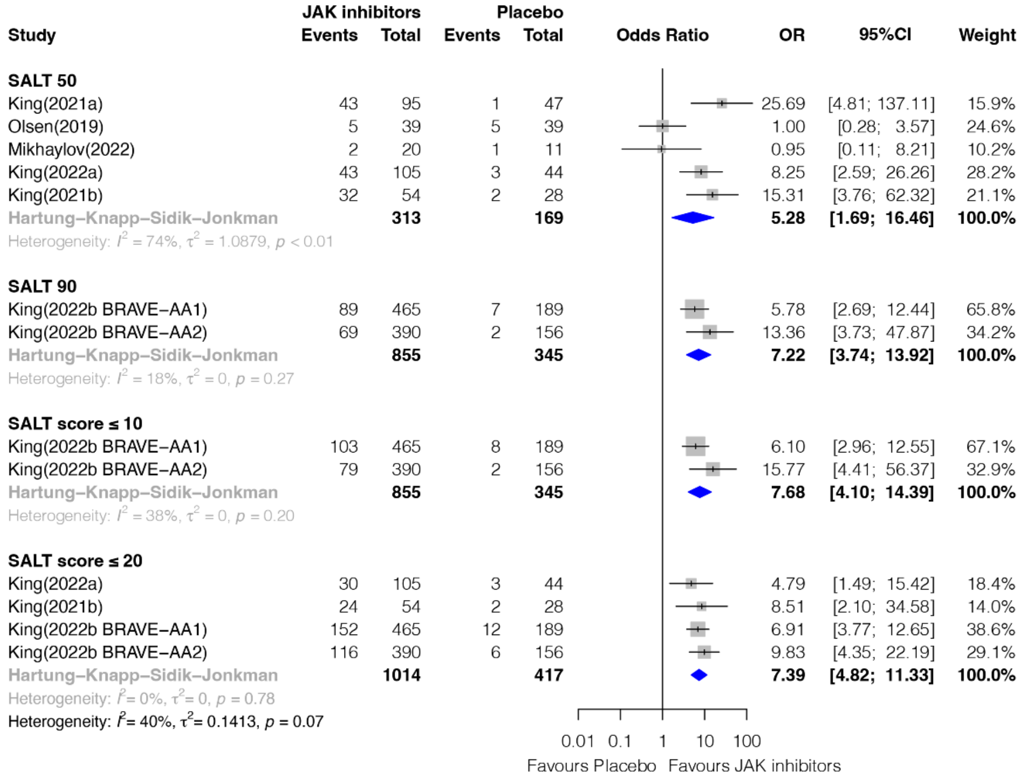

© 2023 Liu M et al. *JAMA Network Open*

eFigure 21 Sensitivity analysis for safety after excluded zero-events studies

Study	JAK inhibitors Events	JAK inhibitors Total	Placebo Events	Placebo Total	Odds Ratio	OR	95%CI	Weight
**Treatment–related adverse event**								
Olsen(2019)	7	39	7	39		1.00	[0.33; 3.07]	4.0%
Mikhaylov(2022)	5	20	2	11		1.35	[0.25; 7.37]	1.8%
King(2022a)	53	105	18	44		1.46	[0.72; 2.96]	10.2%
King(2021b)	40	54	17	28		1.84	[0.71; 4.77]	5.5%
King(2022b BRAVE–AA1)	260	465	97	189		1.20	[0.86; 1.69]	44.3%
King(2022b BRAVE–AA2)	260	390	97	156		1.22	[0.83; 1.79]	34.2%
**Hartung–Knapp–Sidik–Jonkman**		**1073**		**467**		**1.25**	**[1.00; 1.57]**	**100.0%**
Heterogeneity: I^2 = 0%, τ^2 = 0, p = 0.96								
**Severe adverse event**								
King(2022a)	2	105	2	44		0.41	[0.07; 2.46]	13.8%
King(2022b BRAVE–AA1)	9	465	7	189		0.51	[0.19; 1.34]	46.5%
King(2022b BRAVE–AA2)	17	390	4	156		1.59	[0.55; 4.55]	39.7%
**Hartung–Knapp–Sidik–Jonkman**		**960**		**389**		**0.77**	**[0.40; 1.50]**	**100.0%**
Heterogeneity: I^2 = 33%, τ^2 = 0.0003, p = 0.22								
**Discontinuation due to Adverse**								
King(2021a)	4	95	1	47		1.52	[0.23; 10.00]	14.6%
King(2022a)	2	105	3	44		0.29	[0.05; 1.51]	18.7%
King(2022b BRAVE–AA1)	8	465	2	189		1.39	[0.34; 5.77]	25.6%
King(2022b BRAVE–AA2)	10	390	4	156		0.94	[0.31; 2.87]	41.1%
**Hartung–Knapp–Sidik–Jonkman**		**1055**		**436**		**0.89**	**[0.43; 1.83]**	**100.0%**
Heterogeneity: I^2= 0%, τ^2= 0, p = 0.48								
Heterogeneity: I^2= 0%, τ^2= 0, p = 0.71								

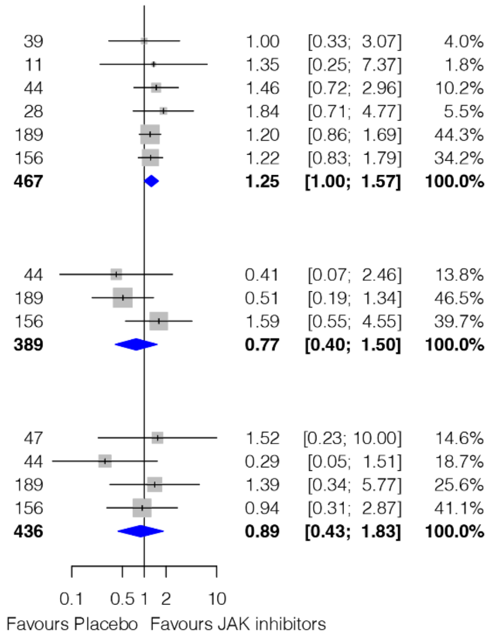

© 2023 Liu M et al. *JAMA Network Open*
