# Supplementary material for: Janus Kinase Inhibitors for Alopecia Areata: A Systematic Review and Meta-Analysis
Source: JAMA Netw Open. 2023 Jun 27;6(6):e2320351. doi: 10.1001/jamanetworkopen.2023.20351 (PMC10300710; doi:10.1001/jamanetworkopen.2023.20351)
Supplement: Supplement 2. — Data Sharing Statement [file jamanetwopen-e2320351-s002.pdf]

Data Sharing Statement

Liu. Janus Kinase Inhibitors for Alopecia Areata. *JAMA Netw Open*. Published June 27, 2023.
doi:10.1001/jamanetworkopen.2023.20351

Data
Data available: Yes
Data types: Deidentified participant data
How to access data: lium2018@lzu.edu.cn
When available: With publication

Supporting Documents
Document types: Statistical/analytic code
How to access documents: lium2018@lzu.edu.cn
When available: With publication

Additional Information
Who can access the data: lium2018@lzu.edu.cn
Types of analyses: any purpose
Mechanisms of data availability: lium2018@lzu.edu.cn
